# Extending health insurance to the poor in India: An impact evaluation of Rashtriya Swasthya Bima Yojana on out of pocket spending for healthcare

**DOI:** 10.1016/j.socscimed.2017.03.053

**Published:** 2017-05

**Authors:** Anup Karan, Winnie Yip, Ajay Mahal

**Affiliations:** a Indian Institute of Public Health Delhi (IIPHD), Public Health Foundation of India, Delhi NCR, India; b Department of Global Health and Population, Harvard T. H. Chan School of Public Health, Harvard University, Boston, USA; c Nossal Institute for Global Health, University of Melbourne, Melbourne, Australia

**Keywords:** India, Health insurance, RSBY, Impact evaluation, Financial burden

## Abstract

India launched the ‘Rashtriya Swasthya Bima Yojana’ (RSBY) health insurance scheme for the poor in 2008. Utilising 3 waves (1999–2000, 2004–05 and 2011–12) of household level data from nationally representative surveys of the National Sample Survey Organisation (NSSO) (N = 346,615) and district level RSBY administrative data on enrolment, we estimated causal effects of RSBY on out-of-pocket expenditure. Using ‘difference-in-differences’ methods on households in matched districts we find that RSBY did not affect the likelihood of inpatient out-of-pocket spending, the *level* of inpatient out of pocket spending or catastrophic inpatient spending. We also do not find any statistically significant effect of RSBY on the level of outpatient out-of-pocket expenditure and the probability of incurring outpatient expenditure. In contrast, the likelihood of incurring any out of pocket spending (inpatient and outpatient) rose by 30% due to RSBY and was statistically significant. Although out of pocket spending levels did not change, RSBY raised household non-medical spending by 5%. Overall, the results suggest that RSBY has been ineffective in reducing the burden of out-of-pocket spending on poor households.

## Highlights

• RSBY has no effects in reducing probability of incurring and level of inpatient expenditure.
• Probability of catastrophic inpatient expenditure declined only marginally.
• Probability of incurring of total out-of-pocket expenditure increased by 30%.
• Non-medical expenditure of households increased by 6% after the RSBY intervention.

## 1 Introduction

In recent years, several developing countries have introduced tax-financed health insurance coverage to their poor populations (Wagstaff et al., 2009, Giedion et al., 2013) India too, joined this effort in 2008, with the Indian Ministry of Labour and Employment (MoL&E) launching the ‘*Rashtriya Swasthya Bima Yojana*’ (RSBY) to protect poor Indian households from financial risks associated with hospitalization expenses. By September 2016, more than 41 million families (about 150 million people) out of a targeted 65 million families, were enrolled in RSBY (RSBY, about, RSBY, documents1, RSBY, documents2).

We assess the impact of RSBY on multiple indicators of financial risk protection among poor Indian families in contrast to existing studies, which have focused on enrolment, service use patterns, patient satisfaction, and implementation barriers in RSBY (Palacios, 2011, Sun, 2011, Rajasekhar et al., 2011, Das and Leino, 2011, Nandi et al., 2013, Hou and Palacios, 2011). Our paper advances the limited literature that has examined financial risk protection among families enrolled in RSBY. For example, Rathi et al., 2012 and Devadasan et al., 2013 found families enrolled in RSBY continued to incur out-of-pocket (OOP) spending, particularly on drugs and diagnostics, during and/or following hospitalization, despite RSBY being a cashless scheme with no co-payment or fees at the point of service. However, the analyses of these studies are based only on data on RSBY enrollees and lacks controls, and thus cannot identify the program effects of RSBY. They were also limited in their geographical scope, covering one district each (RSBY covers 520 out of a total of 625 districts in India). Ghosh (2014) sought to assess financial protection for poor households covered in RSBY in the state of Maharashtra, and concluded that RSBY did not affect household catastrophic health expenditure. Although a control group of households is used, the study's reliance on cross-sectional data implies that RSBY program effects cannot be separated from unobserved confounders. Finally, Selvaraj and Karan (2012) used NSSO OOP data for pre and post intervention periods (2004–5 and 2009–10 respectively) to assess the implications of health insurance programs for the poor. Although they find no beneficial effects of health insurance, their analysis does not specifically assess RSBY (other state-funded insurance programs are in the mix) and does not directly control for observed confounders.

Our paper also contributes to the broader international and Indian literature on the impacts of health insurance programs on household financial risk protection in low- and middle-income countries. According to this literature, increased health insurance coverage has promoted use of health services; but the impacts on financial risk protection are less certain and tend to be context dependent, especially for poor beneficiaries (Escobar et al., 2010, Acharya et al., 2012, Giedion et al., 2013). It has been suggested that the inconclusive results in the existing literature may partly have arisen from inadequate handling of ‘observed’ and ‘unobserved’ heterogeneity, reflected in self-selection of sicker individuals into the insurance schemes, differential health seeking behaviour, and various non-price constraints (Wagstaff, 2007, Acharya et al., 2012, Wagstaff and Lindelow, 2008, Wagstaff et al., 2009, Wagstaff, 2010).

The literature on Indian programs other than RSBY is also limited (Nandi et al., 2015). Some studies (Ranson, 2002, Devadasan et al., 2007, Devadasan et al., 2010) have focused on small-scale community-based health insurance (CBHI) programs, finding that these schemes raise healthcare utilization rates and lower household financial burden. Four recent studies have evaluated relatively large social health insurance schemes in India. Aggarwal (2010) found that the *Yeshasvini* scheme in Karnataka state reduced OOP financed by savings, income and other sources by up to 74% and borrowings by more than 30%. Also in Karnataka, Sood et al. (2014) used a regression discontinuity design across 572 villages to evaluate the *Vajpayee Arogyashree* (VAS) health insurance scheme, finding eligible households experienced reduced OOP health expenditures for hospitalizations. Fan et al. (2012) found that *Rajiv Aarogyasri* (RAS) scheme in the state of Andhra Pradesh reduced inpatient OOP among the enrolled families during ‘Phase I’ of the scheme but had relatively small impacts on outpatient OOP and catastrophic payments. Finally, Rao et al. (2014) evaluated the effect of RAS using a different dataset to Fan et al. (2012), and found that the program led to significant declines in OOP spending and borrowing for financing inpatient care, in rural areas and among poor households. In contrast to these state-level schemes, however, RSBY has been at the national level, although not all states participated in it.

We assess, at the national level, the impact of RSBY on financial risk protection of households using data from 3 waves of cross-sectional household surveys of the National Sample Survey Organisation (NSSO) and district level enrolment information from RSBY records. We exploit the differential roll-out of the scheme across districts to estimate the causal effects of RSBY on a set of OOP related outcome indicators for households using difference-in-differences (DID) methods, in a set of matched districts. We find that the RSBY did not affect the likelihood of a household reporting any inpatient OOP or catastrophic inpatient expenditure. However, the probability of incurring any outpatient OOP expenditure increased by 23%, while conditional on positive outpatient expenditure, the level of outpatient expenditure declined marginally. Overall, we find little evidence of the impact of RSBY on commonly used indicators of financial risk protection based on OOP spending. However, we do find that household non-medical spending increased due to RSBY.

## 2 Background on the RSBY scheme

The Indian Ministry of Labour and Employment (MoL&E) launched the RSBY in April 2008, to provide insurance coverage for inpatient care to poor families (or ‘Below Poverty Line’ [BPL] families). Only households on the BPL list (the list of poor households based on a census conducted by each state) of a state are eligible to enrol in RSBY.

RSBY-covered households are entitled to hospitalization coverage of up to INR 30,000 (approximately US$500) annually for a specified list of conditions. Pre-existing conditions are covered, but outpatient services are not. Coverage is limited to a maximum of five family members. Beneficiaries pay an annual registration fee of INR 30 (approximately US$0.50) per household. The scheme is funded by contributions from the central and state governments and managed by public and private insurance companies, selected via competitive bidding. Covered services under RSBY are delivered by hospitals empanelled under the scheme. Currently, 11 insurance companies (4 public and 7 private) manage the scheme across India, and the number of empanelled service providers (registered with RSBY after meeting the laid down quality criteria) exceeds 10,700 (of which more than 6000 are in the private sector) across India. Table 1 summarizes the main features of the program.

Districts in each state participated in the scheme in a staggered manner. In the first year of RSBY implementation, 20% of all the districts in a state were allowed to participate. In each subsequent year, an additional one-fifth of each state's districts were allowed to participate, subject to availability of adequate numbers of providers, insurance companies and updated lists of poor households. State governments, however, decide whether and when districts can participate in RSBY (Ministry of Labour and Employment, 2008a, Ministry of Labour and Employment, 2008b).

### 2.1 Progress of enrolment

**Fig. 1:**
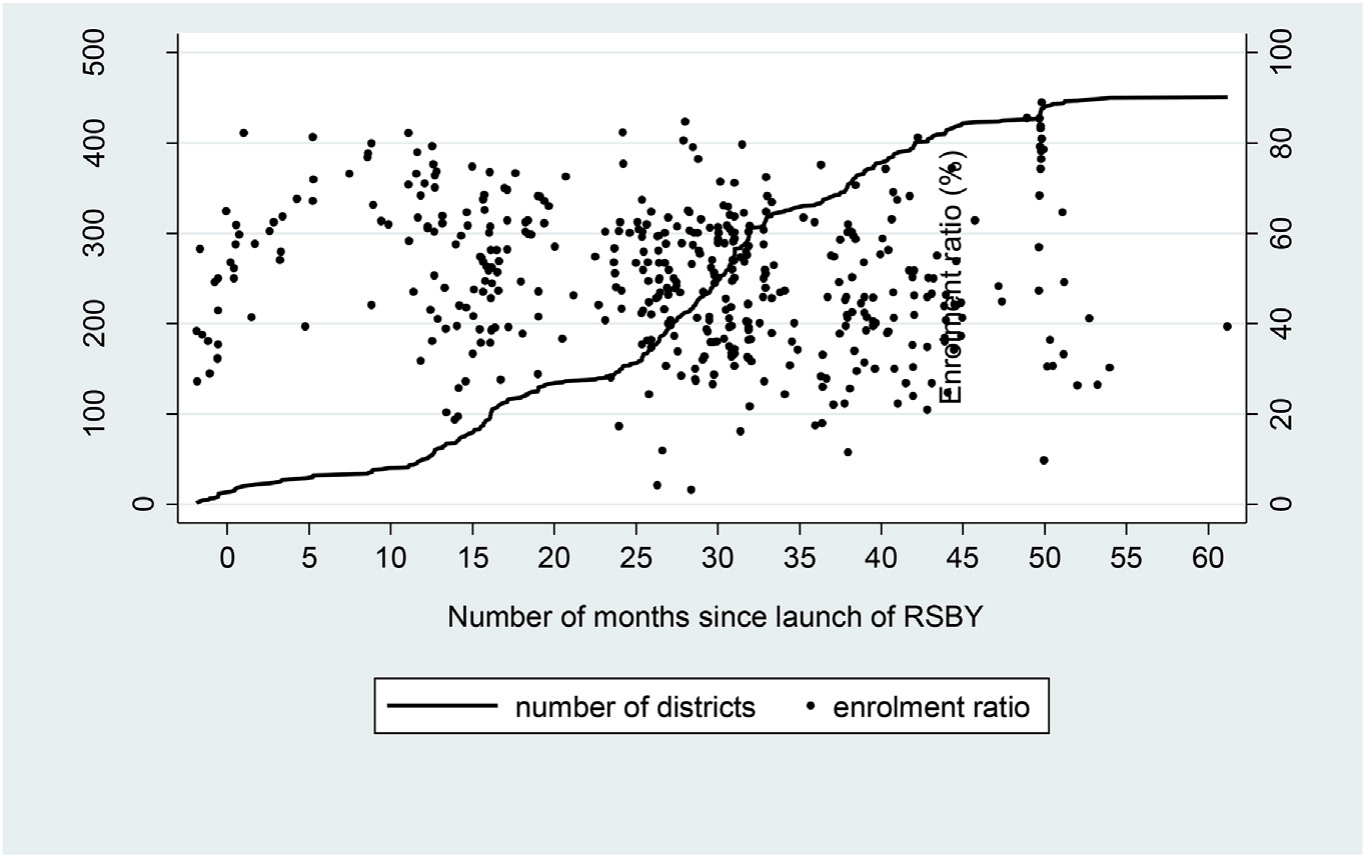
Cumulative number of districts and enrolment ratio (%) as on 31 March 2013.

As of September 2016, more than 41 million health cards (signifying enrolment in RSBY) had been issued, covering almost 150 million poor people, with nearly 460 districts participating in the programme. Although the share of eligible households enrolled in the program (enrolment ratio) was 57% nationally, there was considerable variation across districts, as shown in Fig. 1. Enrolment ratios varied from a low of 3% in Kannauj and 6% in Kanpur Dehat districts in Uttar Pradesh, to nearly 90% in many districts of Chhattisgarh and Kerala. The detailed break-down of number of districts covered under RSBY and range of enrolment ratios in participating states is presented in Appendix Tables A-I and A-II respectively.

Not all states participate in RSBY. Andhra Pradesh did not adopt RSBY as it already provides a generous health insurance scheme (RAS) (Fan et al., 2012). The states of Jammu & Kashmir and Madhya Pradesh are officially participating in the scheme, but as of September 2016, none of their districts had enrolled households into RSBY. In two other states (Karnataka and Tamil Nadu), RSBY has been rolled-out in only a few districts, with other districts being covered by their respective state-financed health insurance schemes (VAS and *Yeshasvini* in Karnataka and Chief Minister Health Insurance Scheme [CMCHIS] in Tamil Nadu). These state-specific schemes provide a more generous benefit package (up to INR 200,000 for hospital services) and cover a broader population group than RSBY. Another state, Rajasthan, was still in the early stages of rolling-out RSBY as of September 2016.

Given our goal of evaluating the impact of RSBY, survey households in the 3 states (Andhra Pradesh, Karnataka and Tamil Nadu) with state specific schemes were dropped from our analysis. Households in the state of Delhi were also dropped from our analysis due to the unavailability of district-level enrolment data. Dropping these states reduced the sample by 65,458 households which comes to about 18% of all the households.

An overall enrolment rate of 57% suggests a large number of uncovered households who are otherwise eligible for RSBY. One possibility is that these households are living in districts that have not participated in RSBY thus far (Palacios et al., 2011, Sun, 2011). Another possibility is that even in participating districts, enrolment agencies may not yet have reached all eligible households (Sun, 2011, Rathi et al., 2012). Finally, eligible households may have simply fallen through the cracks and ended up not getting enrolled (e.g., lack of adequate outreach by enrolment agencies, or absence at the time of enrolment) (Rajasekhar et al., 2011, Sun, 2011, Rathi et al., 2012, Devadasan et al., 2013). Although large scale ‘adverse selection’ in RSBY is unlikely as the scheme is free, households with healthier family members may have less incentive for voluntary RSBY enrolment (Sun, 2011).

### 2.2 Assessing the impact of RSBY: theoretical predictions

The likely impacts of RSBY on health care utilization and OOP payments can be understood in the context of a standard household utility-maximization model, with a health production function and an income constraint, given prices of healthcare services and other goods. Since RSBY reduces the price of inpatient care, households are hypothesized to increase the utilization of inpatient care. RSBY is also likely to reduce the financial burden of inpatient care on households. However, if households are incentivized to use inpatient care beyond the RSBY-sanctioned limit (INR 30,000), inpatient OOP may not decline.

The direction of RSBY's effect on utilization of outpatient services and OOP spending on outpatient care is, however, ambiguous. The impact on outpatient care will depend on whether it complements or substitutes inpatient care. For instance, if the increased inpatient care (because of RSBY) necessitates complementary pre- and post-hospitalization outpatient services, outpatient care use will likely increase. However, if outpatient care substitutes inpatient care, outpatient care use is likely to decline. Because RSBY does not cover outpatient care expenses, its effect on outpatient OOP will be in the same direction as for outpatient care use.

The effect of RSBY on total OOP, and non-medical household consumption expenditure will depend on the net outcome of changes in the inpatient and outpatient OOP as summarised in Table 2. While RSBY will affect household non-medical consumption expenditure, the direction of this effect is uncertain. Because the direction of the effects on OOP spending (inpatient or outpatient) is uncertain, so is non-medical spending which is a measure of household income net of medical spending.

## 3 Methods

### 3.1 Data

The study used data from three waves of household ‘Consumer Expenditure Surveys’ (CES): 1999–2000, 2004–5 and 2011–12, conducted by the NSSO. Sample sizes in each of the 3 rounds ranged between 100,000 and 125,000 households.

The CES collect socioeconomic and demographic information on households, but their major focus is on household spending on roughly 350 food and non-food items. Out-of-pocket medical expenses incurred by households are separately recorded for inpatient and outpatient services. The recall periods are one-year and 30-days for inpatient and outpatient expenses, respectively.

The CES are repeated cross-section surveys and are representative at the national and state levels. In most cases, all districts of a state are included for sampling purposes. Households are sampled evenly in quarterly sub-rounds beginning on 1 July and ending on 30 June of the following year, with equal numbers of households allotted in each quarterly sub-round, to address seasonality. All estimates in the present paper are sample weighted.

In addition to CES, we used district-level information on household enrollments under the RSBY program (http://www.rsby.gov.in/Overview.aspx).

#### 3.1.1 Pre- and post-intervention years

Implementation of the RSBY scheme began in 2008–9. Thus, the 2011–12 wave represents the post-intervention year, the 1999–2000 wave represents the baseline and the 2004–05 wave provides an additional data point for the pre-intervention period. We define these years as: 2000 (‘*t*_*1*_*’*), 2005 (‘*t*_*2*_*’*) and 2012 (‘*t*_*3*_*’*).

#### 3.1.2 Treatment and controls

All eligible (poor) households in RSBY implementing districts are taken as the treatment group, with the poor in non-RSBY districts being the control group. The NSSO data can help identify the BPL status of households in 2004–05 and 2011–12 (our population of interest), but not in 1999–2000. However, a comparison of households’ self-reported BPL status and consumption expenditure per capita in 2004–05 and 2011-12 suggests that the two lowest per capita expenditure quintiles account for about 65 per cent (more than 70 per cent in the RSBY intervention districts) of households with BPL status (Appendix Table A-III). For this reason, we used households belonging to the two poorest expenditure quintiles as a proxy for BPL households. Analogously, the two poorest expenditure quintiles in the non-intervention districts formed the control groups.

#### 3.1.3 Outcome indicators

We used 4 household-level indicators of the financial burden of illness, of which three: per household member monthly OOP spending (inflation-adjusted); OOP spending as a share of household spending; and whether a household reported catastrophic healthcare payments (OOP spending greater than 10% of household consumption expenditure) – were based on OOP payments. Although a variety of thresholds have been used in the literature, the 10% threshold has been commonly used for assessing catastrophic expenditure (Xu et al., 2003, Doorslaer et al., 2007, Fan et al., 2012, Karan et al., 2014) and we used the 10% threshold. We also considered an alternative threshold of 25% for the ratio of OOP to household non-food expenditure, which reflected almost similar results. Expenditure indicators were constructed separately for inpatient care, outpatient care and combined (inpatient care *plus* outpatient care). Our final indicator of financial burden was monthly household non-medical expenditure (households’ total spending – total OOP) per member.

#### 3.1.4 Control variables

Control variables included: indicators of caste (Scheduled Caste [SC], Scheduled Tribe [ST], Other Backward Classes [OBC]); religion (Hindu, Muslim or other); employment status of household (self-employed, regular wage worker, casual wage worker, and others); location (rural or urban); household size, two indicators of household energy use, demographic structure (proportion of persons in different age groups); educational attainment of head of household; the ratio of female to male household members and asset index of households. Summary statistics are available in Appendix Table A-IV.

Ethical approval for this study was not needed. The study used only anonymised data from secondary sources. Requisite permission to use the data has been obtained from the agency.

### 3.2 Empirical strategy

#### 3.2.1 DID procedure

Difference-in-differences (DID) methods, combined with propensity-score matching, were used to evaluate the causal impacts of RSBY on the four outcome measures. DID estimators compare the change in outcomes before and after the intervention between households that have been exposed (treated) to the intervention and households that have not been exposed (untreated). Because all eligible households in RSBY implementing districts, whether enrolled or not, are considered as treated under our identification strategy (outlined below), we estimate an ‘intention to treat’ (ITT) effect, and not the average treatment effect on the treated (ATT) (Imbens and Wooldridge, 2009). The ITT effect can approximate ATT if the ‘insurance uptake’ (enrolment ratio) is high (Miller et al., 2009, Acharya et al., 2012).

#### 3.2.2 Common trend assumption

DID estimation assumes that in the absence of treatment the change in outcome between pre- and post-intervention periods for the treated is similar to the untreated (Abadie, 2008). This assumption may not hold if treated and untreated groups differ in the distribution of their observable and/or unobservable characteristics, and if these characteristics are systematically related to the probability of households being treated. For instance, districts selected for RSBY participation may have better management and implementation capacity. Inability to control for systematic trends in a DID framework will result in under (over) estimation of programme effects (Heckman and Vytlacil, 2007).

We included two pre-intervention years, 2000 and 2005, in the analysis to identify differential pre-intervention trends in outcomes between the RSBY and non-RSBY districts (‘treatment’ and ‘control,’ respectively). In addition, we use propensity score matching methods to create comparable treatment and control districts using pooled data from the two pre-intervention years (2000 and 2005). Propensity scores were generated using a *logit* model with the following explanatory variables: (1) region of location of districts (North, South, East, West and Central India, with North-East states and Union Territories (UTs) as comparison group), (2) share of population that is rural, (3) poverty indicator (proportion holding BPL ration card), (4) share of SC and ST population, (5) share of population that is illiterate and (6) a set of district-level health and social infrastructure indicators. The logit estimates (Appendix Table A-V) suggest that districts in the North-East and UTs are less likely to participate in RSBY than districts in other regions, with districts in central and south India having the highest likelihood of participation compared to others. Districts with a larger share of rural population were more likely to participate, whereas districts with larger shares of poor, SC and ST, and illiterate populations were less likely to participate in the scheme. Among the district level infrastructure, almost all the indicators reflect very marginal difference in the likelihood of participation of districts in RSBY. Restricting the sample districts to observations within the common support range reduced the number of treated and control districts (from 369 to 355 and 166 to 141, respectively). The districts were then matched on the basis of nearest neighbour matching (alternatively stratification method, results not presented). The balancing property and test of balancing is presented in the Appendix Table A-VI and Appendix Table A-VII. In addition, we also conducted household level characteristics matching and performed DID analysis only considering matched households across the three years. Household matching results are presented in Appendix Tables B-I-III. The two different matching strategies reflected almost similar results (compare Table 4 and Appendix Table B-III).

**Fig. 2:**
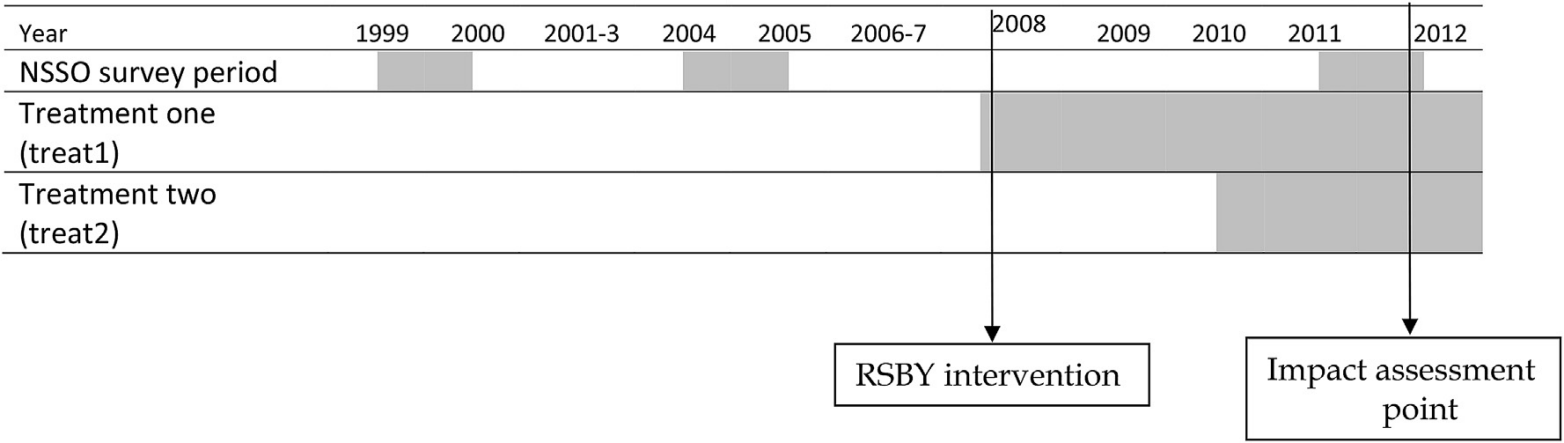
Periods of NSSO data, intervention year and the point of impact assessments.

Because RSBY effects might vary with years of exposure, we considered two discrete cut-off points, March 2010 and March 2012, to identify two treatment groups with differing lengths of programme (RSBY) exposure. These were: i) poor households living in districts which began participating in RSBY on or before March 2010 (‘treat1’—early treatment) and ii) those living in districts which began participating between April 2010 and March 2012 (‘treat2’—late treatment; Fig. 2). The number of districts and the sample households in the two treatment groups and their common control group (before and after matching of districts) is presented in Table 3.

In our DID regression, we control for household level socio-economic observable characteristics and state-level fixed effects (equation (1)). State level fixed-effects were used because policymakers at the state level decide which districts participate in RSBY. The regression specification of DID is as follows:

(1) $$y_{ijt}=\alpha +d_{t}+\beta_{1}treat1+\beta_{2}treat2+\sum_{t=2}^{3}d_{t}\cdot treat1\cdot \phi_{t}+\sum_{t=2}^{3}d_{t}\cdot treat2\cdot \varphi_{t}+\gamma \cdot X_{ijt}+\eta_{j}+\varepsilon_{it}$$

Where, *y*_*ijt*_ is the outcome of interest for household *i* living in district *j* in time period *t*, *d*_*t*_ stand for the time dummies, (*‘t2’* and *‘t3’* for 2005 and 2012, respectively). The terms *‘treat1’* and *‘treat2’* are two dummy variables indicating early and late RSBY intervention districts, respectively.

The two time dummies ‘*d*_*t*_’ (*‘t2’,* and *‘t3’*) are interacted with the two treatment groups separately. To help with interpretation, for the treatment group that joined RSBY on or before March 2010 *(treat1),* ‘*ϕ*_*2*_’ is the pre-intervention DID estimate (comparing 2005 to the baseline year of 2000) and ‘*ϕ*_*3*_’ is the post-intervention DID estimate (comparing 2012 to the base line year of 2000). Similarly, for the treatment group that joined RSBY between March 2010 and March 2012 *(treat2),* ‘*φ*_*2*_'is the pre-intervention DID estimate; ‘*φ*_*3*_'is the post-intervention DID estimate. *X*_*ijt*_ stands for a set of socio-economic covariates for households ‘*i*’ living in district ‘*j*’ in period ‘*t*’. The two error terms represent state level fixed effects ($\eta_{j}$) and an independently distributed error term ($\varepsilon_{it}$). Finally, robust standard errors were clustered at the district level and sampling weights were used.

If ‘*ϕ*_*2*_’ in equation (1) is not significantly different from ‘0’, then the common trend assumption can be taken as satisfied for treat1. In that case, ‘*ϕ*_*3*_ - *ϕ*_*2*_’ provide estimates of the effects of RSBY under the common trend assumption for the treat1 group, as the interaction term coefficients ‘*ϕ*_*2*_’ and ‘*ϕ*_*3*_’ are DID estimates for pre- and post-intervention years, respectively, based on the extended pre-intervention base line year 2000. However, if ‘*ϕ*_*2*_’ is significantly different from ‘0’, the common trend assumption is not satisfied and ‘*ϕ*_*3*_ - *ϕ*_*2*_’ does not provide the actual effects of RSBY (Mora and Reggio, 2012). Similarly, for the treat2 group ‘*φ*_*3*_ - *φ*_*2*_’ provides effects of the scheme only if ‘*φ*_*2*_’ is not significantly different from ‘0’.

Given the large proportion of zeros for our three OOP-related outcome variables in the sample (26% for total OOP, 86% for inpatient OOP and 30% for outpatient OOP), we estimated the DID specification for these outcomes using the ‘two-part-model’ (TPM; Manning et al., 1987), with part one being a ‘logit’ model for estimating a household's probability of incurring OOP; and part two is a specification describing the relationship between (log) monthly OOP incurred by households per person and explanatory variables, conditional on positive payments for using health care. Equation (1) can be specified under the TPM framework as follows:

(2.1) $$\log\;it\left(y_{ijt}\right)=\alpha +d_{t}+\beta_{1}treat1+\beta_{2}treat2+\sum_{t=2}^{3}d_{t}\cdot treat1\cdot \phi_{t}+\sum_{t=2}^{3}d_{t}\cdot treat2\cdot \varphi_{t}+\gamma \cdot X_{ijt}+\eta_{j}+\varepsilon_{it}$$

(2.2) $$\log\left(y_{ijt}|y_{ijt}>0\right)=\alpha +d_{t}+\lambda_{1}treat1+\lambda_{2}treat2+\sum_{t=2}^{3}d_{t}\cdot treat1\cdot \omega_{t}+\sum_{t=2}^{3}d_{t}\cdot treat2\cdot \psi_{t}+\zeta \cdot X_{ijt}+\nu_{j}+\mu_{i}$$

Because the share of OOP in household expenditure ranges between 0 and 1, we estimated Part II of the TPM using simple OLS. For the outcome variable related to catastrophic payment, we estimated a logit model in part I representing the probability of households incurring OOP that exceed the threshold of 10% of household consumption expenditure. For estimating the effect on households’ non-medical expenditure, we used semi-log model (equation (2.2) above).

## 4 Results

### 4.1 Descriptive statistics

Summary statistics for the three sets of outcome indicators for treatment and control households in 2000, 2005 and 2012, with and without matching of districts, are reported in Appendix Table A-VIII. Restricting the sample of households to those in matched districts results in smaller differences in mean pre-intervention outcomes for treatment and control households (both treat1 and treat2). We also find that simple pre-intervention difference-in-differences in mean outcomes remained either unchanged or declined following matching. This lends some support to our strategy of matching the districts prior to applying DID to estimate the effects of RSBY.

### 4.2 DID estimates and effects of RSBY

Our results are presented in Table 6, Table 5, Table 4. For clarity, only the two DID coefficient estimates (separately for the two groups, ‘treat1’ and ‘treat2’) are presented in Table 4, Table 5, Table 6 The coefficient estimates for Part-I of the TPM (logit models) are presented as odds ratios. The odds ratios and coefficient estimates of the early- and late-treatment districts are distinguished by the interaction terms suffixed with ‘treat1’ and ‘treat2’, respectively. In addition, the tables also contain results on the ratio of the odd ratios when a logit model was estimated and the difference in the coefficient estimates in the context of the semi-log model of the immediate pre- and post-intervention DID estimates for the districts ‘treat1’ and ‘treat2’, separately.

#### 4.2.1 Inpatient expenses

The results in Table 4 show that the pre-intervention DID coefficient estimates are not statistically significant for all outcomes of interest. That is, pre-intervention, there are no significant differences in trends of the outcome variables between the treatment and control districts, which provides support to the parallel-trend assumption.

We find that RSBY increased the likelihood of incurring any inpatient OOP in the treatment group ‘treat1’ by 22% relative to controls (ratio of the odd ratio of 1.22). However, this increase is not statistically significant. Also, conditional on having positive inpatient OOP, the household OOP spending per person remained unchanged for the treatment compared to controls. We also find that there is no effect of the scheme on the share of inpatient OOP spending in total household expenditures. Although our results show that for the ‘treat1’ group, RSBY lowers the likelihood of experiencing catastrophic inpatient OOP spending by 26%, the effect is not statistically significant.

In ‘treat2’ districts, RSBY increased the probability of incurring any inpatient OOP by 28% and lowered per member OOP inpatient expenditure (conditional on reporting any inpatient OOP) by 16%, but again these were statistically insignificant. We did not observe any impact of RSBY on inpatient OOP as a share of total household spending in ‘treat2’ households. RSBY also lowered the probability of incurring any catastrophic inpatient OOP by almost 9% in ‘treat2’ households, but this was statistically insignificant.

#### 4.2.2 Outpatient OOP and total OOP expenses

In ‘treat1’ districts, RSBY increased the likelihood of incurring outpatient OOP in treatment households by 23%; and per person outpatient OOP (conditional on reporting any outpatient OOP) declined by 5% in 2012 and these impacts were statistically significant. However, RSBY did not affect the share of outpatient OOP in total spending. The probability of catastrophic outpatient OOP among treat1 households was lower by 11% but remained statistically insignificant. We also find no statistically significant effect of the scheme in the treat2 households, except for per person monthly outpatient OOP spending, which declined by 19%.

Total OOP spending showed mostly statistically insignificant differences in the changes in all the four OOP indicators between treatment and control groups, excepting 30% increase in probability of any OOP payments in treat1 and 11% decline in OOP level among the treat2.

#### 4.2.3 Nonmedical expenditure

We also assessed the implications of RSBY on non-medical expenditure of households. We found that RSBY increased nonmedical expenditure of households in the treat1 group by 5%, but not in the treat2 group of households.

### 4.3 Secondary analyses

#### 4.3.1 Drug and non-drug expenditure

Previous studies have suggested that the effect of RSBY on financial risk protection is minimal because beneficiaries continued spending for drugs, either because drugs are unavailable at empanelled hospitals, or because prescribed drugs are not covered by RSBY (Rathi et al., 2012, Devadasan et al., 2013). We explored whether RSBY had differential effects on drug and non-drug OOP expenses for inpatient services (Table 5). In ‘treat1’ districts, RSBY did not affect the likelihood of incurring both drug and non-drug inpatient OOP. However, conditional on positive non-drug OOP, the level of OOP was 27% higher among treat1 households after RSBY was introduced, and this difference was statistically significant. The results for ‘treat2’ households are mostly small and insignificant.

#### 4.3.2 High enrolment districts

Because the effects of RSBY may be limited by low enrolment rates among eligible households, we replicated our analysis using only data for treated districts with “high enrolment rates,” defined as enrolment exceeding 50% of eligible families (Table 6). We do not find evidence of larger effects in high-enrolment districts. The direction of change of all the outcome indicators remained largely similar to the findings for the broader set of intervention districts, reported in Table 4. Also, these results are likely to be biased on account of sample selection: for example, there might be unobserved reasons why these districts were able to achieve higher enrolment rates and these factors could also be correlated with the performance of RSBY.

#### 4.3.3 Spill over to non-eligible households

The scheme is not expected to affect outcomes for non-eligible households. However, non-eligible households living in intervention districts may realise some spill-over effects of the scheme. To identify any spill over effects on ineligible households living in RSBY intervention districts i.e. non-poor households, equations (2.1), (2.2)) were estimated for the top 40% households defined in per capita consumption expenditure terms, in RSBY intervention and control districts. The controls were the top 40% of households in non-intervention districts. We confined this analysis to households in the high enrolment districts where any effects were likely to be salient. We did not find evidence of spill-over effects.

## 5 Discussion and conclusion

We evaluated the effects of RSBY on OOP payments up to March 2012. The inpatient care utilization rate under the scheme remains low: of the 35.5 million families (approximately 130 million persons) enrolled in RSBY over the period from 2008 to 2013, the scheme funded only about 5.8 million hospitalizations. By contrast, independently available NSS healthcare utilization and expenditure survey data for 2014 suggest an average of 0.4 hospitalizations per person in the poor group; therefore, the covered population should have experienced approximately 50 million hospitalizations annually. Moreover, various field based studies (e.g., Das and Leino, 2011, Rajasekhar et al., 2011) show that even the reported enrolment numbers of 35.5 million by March 2012 are overestimates as many family members in the enrolled households were not actually enrolled.

Overall our analysis shows that RSBY has not provided any significant financial protection for poor households. Drug expenditures do not provide the full story on why these impacts are small. The burden of outpatient expenditures that account for the bulk of OOP healthcare spending is mostly unaffected and utilization of outpatient care may even have increased on account of RSBY.

The question is: if RSBY covered inpatient expenses of the enrolled households up to INR 30,000, why did spending for inpatient care and finally OOP burden not decline for eligible households?

There are several plausible explanations. First, enrolled households may have been persuaded by providers to utilize inpatient services not covered by RSBY, or members of enrolled households may have been denied care by empanelled hospitals. Some studies (e.g., Rajasekhar et al., 2011, Devadasan et al., 2013, Ghosh, 2014) have suggested that many hospitals refuse to admit RSBY-enrolled patients due to administrative concerns such as delayed reimbursement by RSBY to hospitals. Second, the relatively low coverage limit (INR 30,000) of the scheme may have led some households to utilize hospital services beyond the RSBY cap. The survey data indicate that out of all households incurring inpatient OOP, approximately 9 per cent in 2012 reported annual OOP of more than INR 30,000, with average annual expenditure being in the range of INR 75–80 thousand. Third, there is evidence from the field that health service providers ask families to purchase expensive drugs and diagnostics (Devadasan et al., 2013) from elsewhere. Finally, even if probability of inpatient OOP were to decline, inpatient OOP spending constitutes only a small proportion (approximately 20%) of total OOP (Selvaraj and Karan, 2009, Selvaraj and Karan, 2012).

On the other hand, there is some evidence that households experienced an increase in non-medical health spending. This suggests that households benefited, even if the benefit did not take the form of lower OOP spending. One potential channel may be increased opportunities for work (and income) resulting from RSBY, with health insurance coverage providing a safety net for household members to pursue economically productive activities.

The approach used in this paper has obvious limitations. First, what we have are ITT and not ATT estimates. Unfortunately, our data does not allow us to identify RSBY-status at the household level and therefore we estimated the effect on RSBY on the eligible households not on the program enrolees. Second, our criterion for the eligibility of households may also have led some poor households to be classified as non-eligible and lowered RSBY impact estimates. We conducted a sensitivity analysis using self-reported household BPL status using two points of time data and found that the results are similar. Third, there is potential bias due to systematic differences between districts that are selected to participate earlier or later in the programme. We combined Diff-in-Diff with two pre-intervention periods propensity score matching of districts to alleviate bias due to such non-random entry into RSBY at the district level. As a robustness check, we also conducted household level matching for all the three years separately and estimated tipple difference ATT and DID estimates using only matched households. All these alternative method reflected very similar results. Finally, the approach to measuring “catastrophic” OOP spending used in this paper is only one approach among various alternatives, for example, indebtedness due to ill health, or asset sales; but we do not have such data available (e.g., Xu et al., 2003, Xu et al., 2007).

From a policy perspective, further investigation is needed to arrive at concrete suggestions. In the interim though, there may be value to increasing enrolment ratios under the scheme, and among those enrolled, to increase their utilization of benefits. Barring a few districts, enrolment ratios have been less than 50% of eligible households even after 5 years of programme implementation in many states. Policy measures may include scaling up of interventions to educate enrolled households of benefits covered under RSBY and increasing household knowledge about their eligibility for RSBY coverage. Clearly, there may be gains to covering outpatient care, especially if these are driven by chronic conditions some of which can be expensive to treat; this may call for expanding the upper limit of benefits offered under the scheme from its current levels and expanding coverage to include some outpatient services. Significant strengthening of health system at the primary level is also needed.

## Figures and Tables

**Table 1:** Key features of RSBY.

Parameter	Description	Additional comments/caveats
Benefits covered	Cost of hospitalization for 725 + procedures at empanelled hospitals up to INR 30,000 per annum per household INR 100 per admission up to INR 1000 for transport cost per annum per household.	Pre-existing conditions are covered; minimal exclusions; day surgeries covered; outpatient expenditure is not covered
Eligibility criteria	Must be on the official state BPL list Limited to five members of the household including household head, spouse and three dependents	All enrolled members must be present at enrolment to be enrolled; infants are covered through mother
Premium and fees	INR 30 registration fee per household per annum paid by household	Average premium for participating districts is around INR 560, funded by the government
Financing	75%/25% Government of India/state government	The ratio is 90%/10% in Northeast states and Jammu & Kashmir
Policy period	One year from month of enrolment	Enrolment can take place over four months each year and can vary across states
Management	Both public and private insurance companies can bid to work in a district or more than a district recommended by state governments	In each district only one insurance company is finally selected for a particular tear
Service provider	Both public and private providers can apply to join the network of providers empanelled under the scheme	Minimum eligibility criteria on quality of services have been laid down by the MoL&E

**Table 2:** Summary of the predicted effects of RSBY.

	Inpatient	Outpatient	
		Complements of inpatient care	Substitutes of inpatient care
Probability of Use	Increase	Increase	Decrease
Out-of-pocket payment	Decrease or increase	Increase	Decrease
Non-medical expenditure	Decrease or increase	Decrease	Increase

**Table 3:** Reference treatment and control groups with number of districts^a^ and households^b^.

	Treatment group	Control group
**Before matching**		
I. Treat1	Poor households in districts that began participation on or before March 2010 (District - 220; Household- 13,163)	Poor households in all non-participating districts as of March 2012 (District - 166; Household- 7441)
II. Treat2	Poor households in districts that began participating between April 2010 and March 2012 (District - 149; Household- 7399)	Poor population in non-participating districts as of March 2012 (District - 166; Household- 7441)
**After Matching**		
III. Treat1	Poor households in districts that began participation on or before March 2010 (District - 219; Household- 13,058)	Poor households in all non-participating districts as of March 2012 (District - 140; Household- 3516)
IV. Treat2	Poor households in districts that began participating between April 2010 and March 2012 (District - 137; Household- 7020)	Poor population in non-participating districts as of March 2012 (District - 140; Household- 3516)

a number of districts in March 2012.

b number of households in the sample in year 2011–12.

**Table 4:** Effects of RSBY on inpatient, outpatient and total OOP.

	Inpatient				Outpatient				Total OOP			
	Probability of any OOP	OOP Level (INR)	OOP Share	Probability of Catastrophic	Probability of any OOP	OOP Level (INR)	OOP Share	Probability of Catastrophic	Probability of any OOP	OOP Level (INR)	OOP Share	Probability of Catastrophic
**‘treat1’ Districts**												
t2_treat1	1.033	0.039	0.009*	1.420	0.815	0.092	0.003	0.922	0.850	0.018	0.003	0.985
SE	0.2519	0.182	0.0055	0.404	0.0991	0.060	0.0027	0.143	0.100	0.071	0.0029	0.1526
t3_treat1	1.262	0.045	0.002	1.055	0.999	0.043	0.000	0.821	1.104	−0.014	−0.001	0.878
SE	0.3102	0.158	0.0068	0.266	0.1601	0.061	0.0027	0.122	0.181	0.066	0.0027	0.1109
*Ratio of odds ratios or differences in pre- and post-intervention DID coefficients*												
t3_treat1- t2_treat1	1.223	0.005	−0.007	0.743	1.226^*^	−0.049	−0.004	0.891	1.298*	−0.032	−0.004	0.891
SE	0.2777	0.2120	0.0079	0.2272	0.1806	0.0580	0.0028	0.1425	0.2013	0.0576	0.0029	0.1322
**‘treat2’ Districts**												
t2_treat2	0.792	0.410**	0.012**	1.726	0.826	0.157**	0.005	1.032	0.811	0.140	0.006	1.154
SE	0.2178	0.182	0.0055	0.5667	0.1056	0.073	0.0033	0.211	0.104	0.083	0.0034	0.2301
t3_treat2	1.014	0.247	0.004	1.572	0.903	0.006	0.002	1.036	0.894	0.027	0.003	1.171
SE	0.2699	0.163	0.0070	0.4949	0.1510	0.069	0.0030	0.195	0.154	0.074	0.0030	0.1937
*Ratio of odds ratios or differences in pre- and post-intervention DID coefficients*												
t3_treat1- t2_treat1	1.281	−0.164	−0.008	0.911	1.093	−0.151	−0.004	1.003	1.102	−0.113*	−0.004	1.016
SE	0.3201	0. .2175	0.0081	0.3162	0.1737	0.0735	0.0033	0.1972	0.1788	0.0738	0.0035	0.1879

R-2/pseudo-R2	0.077	0.33	0.154	0.069	0.095	0.140	0.075	0.054	0.086	0.155	0.084	0.066
Observations	83,976	10,689	10,689	83,976	83,976	53,123	53,123	83,976	83,976	57,435	57,435	83,976

Notes: 1. * significant at 10% level; ** significant at 5% level; *** significant at 1% level; 2. standard errors in are mentioned in the second row against each co-efficient/odds ratio; 3. Standard errors clustered at village level; 4. Values in the probability columns are odds ratios of the probabilities of incurring any OOP and catastrophic payments and under OOP level and OOP share are coefficients of per person monthly OOP and OOP expenditure as a share of households’ total consumption expenditure respectively. 5. Values under OOP share should be multiplied with 100 to read in percentage terms.

**Table 5:** Effects of RSBY on inpatient drug and non-drug OOP.

	Drug OOP		Non-drug OOP	
	Probability of any OOP	OOP Level (INR)	Probability of any OOP	OOP Level (INR)
‘treat1’Districts				
t2_treat1	1.067	−0.048	1.356	−0.138
	0.281	0.177	0.302	0.195
t3_treat1	1.149	−0.012	1.297	0.130
	0.282	0.136	0.269	0.241
*Ratio of odds ratios or differences in pre- and post-intervention DID coefficients*				
t3_treat1- t2_treat1	1.077	0.035	0.957	0.268*
	0.2557	0.1833	0.1543	0.2178
‘treat2’ Districts				
t2_treat2	0.889	0.331	1.039	0.253
	0.260	0.174	0.283	0.227
t3_treat2	1.062	0.270	1.093	0.178
	0.279	0.137	0.283	0.267
*Ratio of odds ratios or differences in pre- and post-intervention DID coefficients*				
t3_treat2- t2_treat2	1.196	−0.061	1.052	−0.076
	0.3122	0.1869	0.2042	0.2531
R-2/pseudo-R2	0.072	0.212	0.081	0.284
Observations	83,976	9992	83,976	7504

Notes: 1. * significant at 10% level; ** significant at 5% level; *** significant at 1% level; 2. standard errors in are mentioned in the second row against each co-efficient/odds ratio; 3. Standard errors clustered at village level; 4. Values in the probability columns are odds ratios of the probabilities of incurring any OOP and under OOP level are coefficients of per person monthly OOP respectively.

**Table 6:** Effects of RSBY on inpatient, outpatient and total OOP in high enrolment districts.

	Inpatient				Outpatient				Total OOP			
	Probability of any OOP	OOP Level (INR)	OOP Share	Probability of Catastrophic	Probability of any OOP	OOP Level (INR)	OOP Share	Probability of Catastrophic	Probability of any OOP	OOP Level (INR)	OOP Share	Probability of Catastrophic
**‘treat1’ Districts**												
t2_treat1	1.033	0.091	0.007	1.482	0.849	0.172**	0.007**	1.061	0.808	0.138	0.008**	1.120
SE	0.3005	0.1922	0.0058	0.4497	0.1284	0.0663	0.0030	0.1759	0.1077	0.0829	0.0034	0.1842
t3_treat1	1.159	0.054	0.000	1.049	0.965	0.120*	0.004	0.973	0.991	0.089	0.005	1.015
SE	0.3307	0.1730	0.0071	0.2844	0.1780	0.0630	0.0030	0.1540	0.1870	0.0736	0.0030	0.1360
*Ratio of odds ratios or differences in pre- and post-intervention DID coefficients*												
t3_treat1- t2_treat1	1.122	−0.037	−0.007	0.708	1.137	−0.052	−0.003	0.917	1.22,626	−0.049	−0.004	0.906
SE	0.2675	0.2115	0.0082	0.2283	0.1855	0.0648	0.0032	0.1557	0.2140	0.0647	0.0035	0.1424
**‘treat2’ Districts**												
t2_treat2	0.670	0.464**	0.015**	1.746	0.792	0.137*	0.006	1.081	0.731*	0.135	0.007*	1.218
SE	0.2100	0.1876	0.0060	0.6122	0.1124	0.0793	0.0038	0.2563	0.0989	0.0940	0.0039	0.2746
t3_treat2	0.880	0.280*	0.005	1.605	0.956	−0.021	0.001	1.080	0.885	0.029	0.003	1.236
SE	0.2590	0.1681	0.0073	0.5514	0.1704	0.0739	0.0033	0.2346	0.1656	0.0816	0.0033	0.2347
*Ratio of odds ratios or differences in pre- and post-intervention DID coefficients*												
t3_treat1- t2_treat1	1.313	−0.185	−0.010	0.919	1.208	−0.158*	−0.004	0.999	1.2102	−0.106	−0.004	1.015
SE	0.3521	0.2228	0.0087	0.337	0.2115	0.0849	0.0038	0.2123	0.2128	0.0857	0.004	0.2037

R-2/pseudo-R2	0.083	0.362	0.164	0.078	0.091	0.16	0.089	0.060	0.085	0.179	0.103	0.079
Observations	53,401	7357	7357	53,401	53,401	32,458	32,458	53,401	53,401	35,548	35,548	53,401

Notes: same as in Table 4.
