# Supplementary material for: Extending health insurance to the poor in India: An impact evaluation of Rashtriya Swasthya Bima Yojana on out of pocket spending for healthcare
Source: Soc Sci Med. 2017 May;181:83–92. doi: 10.1016/j.socscimed.2017.03.053 (PMC5408909; doi:10.1016/j.socscimed.2017.03.053)
Supplement: Appendix A [file mmc1.docx]

Appendix Table A-I: Percentage distribution of number of districts by enrolment ranges in percent

|  | Up to March 2010 | | | | Up to March 2012 | | | | Up to March 2016 | | | |
| --- | --- | --- | --- | --- | --- | --- | --- | --- | --- | --- | --- | --- |
| Major states | <25 | >25 to 50 | >50 to 75 | >75 | <25 | >25 to 50 | >50 to 75 | >75 | <25 | >25 to 50 | >50 to 75 | >75 |
| Assam | 20.0 | 20.0 | 60.0 | 0.0 | 20.0 | 20.0 | 60.0 | 0.0 | 0.0 | 13.0 | 87.0 | 0.0 |
| Bihar | 10.0 | 36.7 | 53.3 | 0.0 | 2.6 | 34.2 | 60.5 | 2.6 | 7.9 | 34.2 | 57.9 | 0.0 |
| Chhattisgarh | 6.3 | 37.5 | 50.0 | 6.3 | 0.0 | 22.2 | 61.1 | 16.7 | 0.0 | 0.0 | 0.0 | 100.0 |
| Gujarat | 0.0 | 4.0 | 84.0 | 12.0 | 0.0 | 42.3 | 57.7 | 0.0 | 0.0 | 65.4 | 34.6 | 0.0 |
| Haryana | 5.3 | 47.4 | 26.3 | 21.1 | 4.8 | 66.7 | 28.6 | 0.0 | 19.1 | 61.9 | 19.1 | 0.0 |
| Himachal | 0.0 | 0.0 | 8.3 | 91.7 | 0.0 | 0.0 | 16.7 | 83.3 | 0.0 | 25.0 | 75.0 | 0.0 |
| Jharkhand | 6.7 | 20.0 | 73.3 | 0.0 | 8.3 | 45.8 | 37.5 | 8.3 | 0.0 | 66.7 | 33.3 | 0.0 |
| Karnataka | 0.0 | 0.0 | 0.0 | 0.0 | 0.0 | 0.0 | 0.0 | 0.0 | 0.0 | 13.3 | 76.7 | 10.0 |
| Kerala | 0.0 | 14.3 | 35.7 | 50.0 | 0.0 | 7.1 | 42.9 | 50.0 | 0.0 | 0.0 | 0.0 | 100.0 |
| Maharashtra | 13.8 | 41.4 | 44.8 | 0.0 | 9.7 | 41.9 | 48.4 | 0.0 | 0.0 | 0.0 | 0.0 | 0.0 |
| Orissa | 0.0 | 0.0 | 80.0 | 20.0 | 4.0 | 24.0 | 72.0 | 0.0 | 0.0 | 3.3 | 66.7 | 30.0 |
| Punjab | 5.9 | 58.8 | 29.4 | 5.9 | 0.0 | 50.0 | 45.0 | 5.0 | 0.0 | 45.5 | 45.5 | 9.1 |
| Rajasthan | 0.0 | 0.0 | 0.0 | 0.0 | 0.0 | 0.0 | 0.0 | 0.0 | 0.0 | 6.1 | 57.6 | 36.4 |
| Uttar Pradesh | 20.3 | 23.2 | 55.1 | 1.5 | 11.3 | 69.0 | 18.3 | 1.4 | 51.6 | 41.9 | 6.5 | 0.0 |
| Uttaranchal | 0.0 | 41.7 | 58.3 | 0.0 | 0.0 | 15.4 | 76.9 | 7.7 | 0.0 | 100.0 | 0.0 | 0.0 |
| West Bengal | 0.0 | 16.7 | 58.3 | 25.0 | 16.7 | 11.1 | 44.4 | 27.8 | 0.0 | 47.6 | 47.6 | 4.8 |
| Other NE states | 23.5 | 23.5 | 17.7 | 35.3 | 2.5 | 25.0 | 40.0 | 32.5 | 0.0 | 18.7 | 62.4 | 18.9 |
| UTs | 0.0 | 0.0 | 100.0 | 0.0 | 0.0 | 0.0 | 100.0 | 0.0 | 0.0 | 0.0 | 0.0 | 0.0 |
| All participating states | 10.1 | 27.5 | 49.7 | 12.8 | 5.6 | 39.0 | 43.8 | 11.7 | 5.8 | 31.4 | 44.2 | 18.6 |

Source: [http://www.rsby.gov.in/Overview.aspx. accessed in July 2010](http://www.rsby.gov.in/Overview.aspx.%20accessed%20in%20July%202010), July 2012 and July 2016.

Appendix Table A-II: Total number of districts covered under the RSBY and the average enrolment ratios* in the participating districts during March 2010, March 2012 and March 2013.

| **Participating States** | **All**  **districts** | **Up to March 2010** | | **Up to March 2012** | | **Up to September 2016** | |
| --- | --- | --- | --- | --- | --- | --- | --- |
|  |  | **Number of participating districts** | **Average enrolment ratio (%)** | **Number of participating districts** | **Average enrolment ratio (%)** | **Number of participating districts** | **Average enrolment ratio (%)** |
| Andhra Pradesh | 23 | 0 | 0 | 0 | 0 | 23 | 0 |
| Assam | 26 | 4 | 43.61 | 5 | 43.62 | 23 | 60.12 |
| Bihar | 38 | 18 | 52.46 | 38 | 53.65 | 38 | 51.92 |
| Chhattisgarh | 16 | 16 | 49.43 | 18 | 62.72 | 27 | 92.74 |
| Delhi | 7 | 7 | N.A. | N.A. | N.A. | N.A. | N.A. |
| Goa | 2 | 0 | 0 | 0 | 0 | 0 | 0 |
| Gujarat | 26 | 9 | 64.70 | 26 | 51.94 | 26 | 46.06 |
| Haryana | 21 | 19 | 53.93 | 21 | 43.53 | 21 | 36.92 |
| Himachal Pradesh | 12 | 11 | 84.16 | 12 | 81.19 | 12 | 53.69 |
| Jammu & Kashmir | 14 | 0 | 0 | 0 | 0 | 0 | 0 |
| Jharkhand | 24 | 7 | 52.17 | 24 | 48.33 | 24 | 47.38 |
| Karnataka | 30 | 0 | 0 | 30 | 36.25 | 30 | 60.13 |
| Kerala | 14 | 14 | 89.17 | 14 | 71.89 | 14 | 89.88 |
| Madhya Pradesh | 48 | 0 | 0 | 0 | 0 | 0 | 0 |
| Maharashtra | 34 | 29 | 43.64 | 31 | 46.09 | 0 | 0 |
| Orissa | 30 | 5 | 67.86 | 17 | 57.24 | 30 | 73.02 |
| Punjab | 22 | 17 | 45.04 | 20 | 50.99 | 22 | 53.42 |
| Rajasthan | 32 | 0 | 0 | 0 | 0 | 33 | 71.80 |
| Tamil Nadu | 30 | 0 | 0 | 0 | 0 | 2 |  |
| Uttar Pradesh | 75 | 67 | 46.84 | 74 | 38.62 | 75 | 29.91 |
| Uttaranchal | 15 | 5 | 54.31 | 13 | 54.99 | 13 | 39.22 |
| West Bengal | 19 | 5 | 63.85 | 17 | 56.04 | 21 | 53.21 |
| Other NE states | 54 | 8 | 55.37 | 38 | 62.43 | 52 | 50.27 |
| UTs | 12 | 1 | 50.82 | 1 | 50.82 | 1 | 62.65 |
| All districts | 625 | 235 | 54.50 | 369 | 52.30 | 487 | 57.85 |

Notes: 1. * Enrolment ratio is defined as percentage of households enrolled to total eligible households. Other ‘NE’ states include the North-East states of Arunachal Pradesh, Manipur, Meghalaya, Mizoram, Nagaland, and Tripura. 2. ‘UTs’ stands for Union Territories of Andaman and Nicobar Islands, Chandigarh, Lakshadweep, Dadra & Nagar Haveli, Daman & Diu and Pondicherry; 2. N.A.: Not available. Appendix

Appendix Table A-III Percentage distribution of BPL households by quintile groups of per capita total monthly household expenditure, 2004-05

|  | **Quintile groups of per capita total household consumption expenditure** | | | | | |
| --- | --- | --- | --- | --- | --- | --- |
| **Major states** | **Q1** | **Q2** | **Q3** | **Q4** | **Q5** | **Total** |
| Delhi | 84.10 | 7.48 | 8.42 | 0 | 0 | 100 |
| Haryana | 37.34 | 26.42 | 22.84 | 10.27 | 3.13 | 100 |
| Himachal | 47.39 | 22.75 | 18.56 | 8.27 | 3.04 | 100 |
| J&K | 44.25 | 30.25 | 12.58 | 7.83 | 5.09 | 100 |
| Punjab | 42.06 | 32.59 | 13.77 | 6.95 | 4.62 | 100 |
| Rajasthan | 39.41 | 24.35 | 18.62 | 10.50 | 7.13 | 100 |
| Uttaranchal | 41.94 | 27.60 | 16.03 | 9.77 | 4.66 | 100 |
| Assam | 40.43 | 25.53 | 18.08 | 12.33 | 3.64 | 100 |
| Chhattisgarh | 30.30 | 20.28 | 22.69 | 17.78 | 8.95 | 100 |
| Madhya Pradesh | 34.48 | 25.43 | 21.57 | 13.53 | 4.99 | 100 |
| Uttar Pradesh | 36.78 | 21.25 | 19.45 | 12.97 | 9.56 | 100 |
| Bihar | 29.89 | 23.99 | 22.81 | 16.28 | 7.03 | 100 |
| Jharkhand | 29.49 | 27.38 | 19.05 | 16.55 | 7.53 | 100 |
| West Bengal | 33.00 | 28.99 | 19.08 | 15.10 | 3.82 | 100 |
| Orissa | 31.53 | 26.06 | 20.40 | 16.43 | 5.58 | 100 |
| Maharashtra | 40.74 | 29.50 | 18.64 | 8.72 | 2.40 | 100 |
| Goa | 52.49 | 26.00 | 10.23 | 4.90 | 6.37 | 100 |
| Gujarat | 38.92 | 28.48 | 23.18 | 8.23 | 1.18 | 100 |
| Andhra Pradesh | 26.61 | 26.24 | 21.37 | 17.06 | 8.72 | 100 |
| Karnataka | 34.96 | 25.91 | 21.60 | 14.07 | 3.46 | 100 |
| Kerala | 35.47 | 24.53 | 20.76 | 13.22 | 6.02 | 100 |
| Tamilnadu | 31.66 | 26.95 | 20.78 | 13.81 | 6.81 | 100 |
| Other NE states | 43.63 | 23.21 | 16.10 | 11.95 | 5.11 | 100 |
| All India | 34.03 | 26.04 | 20.45 | 13.69 | 5.80 | 100 |

*Source:* NSSO, 2004-05, CES

Appendix Table A-IV: Mean and standard deviations of different socio-economic characteristics of households

| **Variable** | **1999-2000** | | **2004-05** | | **2011-12** | |
| --- | --- | --- | --- | --- | --- | --- |
|  | **Mean** | **SD Dev.** | **Mean** | **SD Dev.** | **Mean** | **SD Dev.** |
| caste1 (ST) | 0.0888 | 0.2845 | 0.0877 | 0.2829 | 0.0892 | 0.2851 |
| caste2 (SC) | 0.1925 | 0.3943 | 0.197 | 0.3978 | 0.1902 | 0.3925 |
| caste3 (OBC) | 0.3522 | 0.4777 | 0.4015 | 0.4902 | 0.4308 | 0.4952 |
| caste4 (Other) | 0.3664 | 0.4818 | 0.3137 | 0.464 | 0.2898 | 0.4537 |
|  |  |  |  |  |  |  |
| edu1 (Illiterate) | 0.3168 | 0.4652 | 0.2253 | 0.4178 | 0.173 | 0.3782 |
| edu2 (Primary) | 0.1428 | 0.3499 | 0.161 | 0.3675 | 0.1174 | 0.3219 |
| edu3 (Middle) | 0.1921 | 0.394 | 0.2274 | 0.4192 | 0.1945 | 0.3958 |
| edu4 (Higher secondary) | 0.2408 | 0.4276 | 0.2666 | 0.4422 | 0.3534 | 0.478 |
| edu5 (Graduate and above) | 0.1074 | 0.3097 | 0.1196 | 0.3245 | 0.1617 | 0.3682 |
|  |  |  |  |  |  |  |
| religion1 (Hindu) | 0.8335 | 0.3725 | 0.8338 | 0.3722 | 0.8317 | 0.3741 |
| religion2 (Muslim) | 0.1089 | 0.3115 | 0.1124 | 0.3158 | 0.1181 | 0.3228 |
| religion 3 (Christian) | 0.0272 | 0.1626 | 0.024 | 0.1532 | 0.0245 | 0.1547 |
| religion 4 (Other) | 0.0304 | 0.1718 | 0.0298 | 0.1699 | 0.0256 | 0.1579 |
|  |  |  |  |  |  |  |
| % households using safe energy for cooking | 0.242 | 0.4283 | 0.259 | 0.4381 | 0.341 | 0.4742 |
| % households using safe energy for lighting | 0.594 | 0.4910 | 0.653 | 0.4761 | 0.800 | 0.4001 |
|  |  |  |  |  |  |  |
| Household size<5 | 0.6634 | 0.4726 | 0.6913 | 0.462 | 0.7433 | 0.4368 |
| Proportion female | 0.4761 | 0.2056 | 0.4791 | 0.2047 | 0.4767 | 0.2108 |
| Proportion married | 0.4659 | 0.2564 | 0.4751 | 0.2606 | 0.4903 | 0.2672 |
|  |  |  |  |  |  |  |
| Age 0-4 years | 0.0928 | 0.1395 | 0.0863 | 0.1379 | 0.0693 | 0.1274 |
| Age 5-14 years | 0.213 | 0.2182 | 0.2027 | 0.2185 | 0.1816 | 0.2137 |
| Age 15-29 years | 0.2725 | 0.2539 | 0.2729 | 0.2559 | 0.2796 | 0.2657 |
| Age 30-59 years | 0.3341 | 0.2352 | 0.3474 | 0.2375 | 0.37 | 0.2457 |
| Age 60 years and above | 0.0876 | 0.1981 | 0.0907 | 0.2051 | 0.0994 | 0.2181 |
|  |  |  |  |  |  |  |
| Self-employed non-agriculture) | 0.1898 | 0.3921 | 0.2166 | 0.4119 | 0.218 | 0.4129 |
| Self-employed agriculture) | 0.2431 | 0.4289 | 0.2574 | 0.4372 | 0.2366 | 0.425 |
| Wage paid labour (regular) | 0.1126 | 0.3161 | 0.1123 | 0.3158 | 0.1911 | 0.3932 |
| Wage paid labour (casual) | 0.3374 | 0.4728 | 0.3033 | 0.4597 | 0.2766 | 0.4473 |
| Others | 0.1172 | 0.3217 | 0.1104 | 0.3134 | 0.0777 | 0.2677 |
|  |  |  |  |  |  |  |
| Rural | 0.7279 | 0.445 | 0.725 | 0.4465 | 0.6875 | 0.4635 |
| Asset_index | 0.04887 | 0.0485 | 0.1247 | 0.0962 | 0.3027 | 0.133 |
| Number of observations | 120297 |  | 124644 |  | 101662 |  |

Source: NSSO various years.

Appendix Table A-V: Logit results of matching of districts for treat1 and treat2 groups

|  | **treat1*** | | | **treat2*** | | |
| --- | --- | --- | --- | --- | --- | --- |
|  | Odds ratio | Std. Err. | P>z | Odds ratio | Std. Err. | P>z |
| Region North | 1.616 | 0.4515 | 0.086 | 2.171 | 0.8350 | 0.044 |
| Region West | 0.736 | 0.2500 | 0.366 | 0.485 | 0.1795 | 0.051 |
| Region East | 10.475 | 4.6874 | 0 | 18.602 | 8.1032 | 0 |
| Region South | 0.053 | 0.0374 | 0 | 1.000 | (omitted)** | |
| Region Central | 1.675 | 0.5378 | 0.108 | 0.021 | 0.0131 | 0 |
| % Households ration card holder | 0.460 | 0.3075 | 0.245 | 4.028 | 3.2588 | 0.085 |
| % Rural population | 7.172 | 4.4412 | 0.001 | 0.300 | 0.2461 | 0.142 |
| % SC/ST population | 0.279 | 0.1206 | 0.003 | 2.659 | 1.1498 | 0.024 |
| % Illiterate population | 0.313 | 0.1843 | 0.049 | 0.222 | 0.1402 | 0.017 |
| Primary middle school per lakh population | 0.970 | 0.0136 | 0.03 | 0.994 | 0.0157 | 0.688 |
| Health sub-centre per lakh population | 1.004 | 0.0100 | 0.68 | 1.002 | 0.0120 | 0.867 |
| Primary health centre per lakh population | 1.019 | 0.0112 | 0.089 | 0.999 | 0.0099 | 0.917 |
| Government facility per lakh population | 0.989 | 0.0093 | 0.238 | 0.984 | 0.0123 | 0.19 |
| Doctors per lakh population | 1.047 | 0.0063 | 0 | 1.028 | 0.0082 | 0.001 |
| % children with full immunisation | 1.034 | 0.0064 | 0 | 1.002 | 0.0074 | 0.77 |
| % children with no immunisation | 0.995 | 0.0106 | 0.648 | 0.992 | 0.0131 | 0.544 |
| Year 2005 | 4.283 | 1.6029 | 0 | 1.091 | 0.4499 | 0.832 |
| Constant | 0.320 | 0.4661 | 0.434 | 5.415 | 8.7779 | 0.297 |
| pseudo R2 | 0.27 | | | 0.38 | | |
| Number of observations | 679 | | | 511 | | |

Note: * treat1 and teat2 are two sets of districts with early and late interventions. For details see method section; ** no observation for treat2 in this region

Appendix Table A-VI: Balancing test in matched sample for treat1

| Variable | Sample | Treated | Controls | Difference | S.E. | T-stat |
| --- | --- | --- | --- | --- | --- | --- |
|  |  |  |  |  |  |  |
| outcome1 | Unmatched | 0.673 | 0.571 | 0.102 | 0.0163 | 6.24 |
|  | ATT | 0.673 | 0.591 | 0.082 | 0.0293 | 2.8 |

| Variable | Unmatched/  Matched | Mean  Treated | Mean  Control | %  bias | %reduct  bias | t | p>t | V(T)/V(C) |
| --- | --- | --- | --- | --- | --- | --- | --- | --- |
|  |  |  |  |  |  |  |  |  |
| Region North | U | 0.57524 | 0.39326 | 37 |  | 4.7 | 0 | . |
|  | M | 0.57524 | 0.55583 | 3.9 | 89.3 | 0.56 | 0.574 | . |
| Region West | U | 0.17233 | 0.26592 | -22.7 |  | -2.94 | 0.003 | . |
|  | M | 0.17233 | 0.15534 | 4.1 | 81.8 | 0.66 | 0.511 | . |
| Region East | U | 0.13592 | 0.03745 | 35.5 |  | 4.28 | 0 | . |
|  | M | 0.13592 | 0.13835 | -0.9 | 97.5 | -0.1 | 0.919 | . |
| Region South | U | 0.06796 | 0.05618 | 4.9 |  | 0.61 | 0.539 | . |
|  | M | 0.06796 | 0.01699 | 21.1 | -332.6 | 3.65 | 0 | . |
| Region Central | U | 0.37136 | 0.3221 | 10.3 |  | 1.31 | 0.19 | . |
|  | M | 0.37136 | 0.44417 | -15.3 | -47.8 | -2.13 | 0.033 | . |
| % Households ration card holder | U | 0.1691 | 0.1721 | -1.4 |  | -0.18 | 0.86 | 0.91 |
|  | M | 0.1691 | 0.18005 | -5 | -265.2 | -0.73 | 0.468 | 0.93 |
| % rural population | U | 0.87376 | 0.8708 | 1.7 |  | 0.23 | 0.82 | 0.51* |
|  | M | 0.87376 | 0.89086 | -10 | -476.3 | -1.6 | 0.111 | 0.73* |
| % SC/ST population | U | 0.39339 | 0.48178 | -35.4 |  | -4.65 | 0 | 0.55* |
|  | M | 0.39339 | 0.45477 | -24.6 | 30.6 | -3.64 | 0 | 0.61* |
| % illiterate population | U | 0.52937 | 0.58529 | -26.5 |  | -3.37 | 0.001 | 1.03 |
|  | M | 0.52937 | 0.54091 | -5.5 | 79.4 | -0.73 | 0.464 | 0.79* |
| Primary middle school per lakh population | U | 95.264 | 96.086 | -10.9 |  | -1.4 | 0.163 | 0.92 |
|  | M | 95.264 | 96.255 | -13.2 | -20.5 | -1.98 | 0.048 | 1.11 |
| Health sub-centre per lakh population | U | 40.479 | 40.028 | 2.3 |  | 0.29 | 0.776 | 1.55* |
|  | M | 40.479 | 39.681 | 4 | -76.8 | 0.57 | 0.569 | 1.40* |
| Primary health centre per lakh population | U | 15.933 | 12.965 | 17.1 |  | 2.09 | 0.037 | 2.36* |
|  | M | 15.933 | 16.606 | -3.9 | 77.3 | -0.5 | 0.619 | 1.27* |
| Government facility per lakh population | U | 47.484 | 48.047 | -2.7 |  | -0.33 | 0.738 | 1.19 |
|  | M | 47.484 | 47.713 | -1.1 | 59.4 | -0.15 | 0.877 | 1.19 |
| doctor per lakh population | U | 41.577 | 25.663 | 58.9 |  | 7.32 | 0 | 1.60* |
|  | M | 41.577 | 41.11 | 1.7 | 97.1 | 0.22 | 0.825 | 0.97 |
| % children full immunised | U | 53.042 | 40.122 | 55.1 |  | 7.01 | 0 | 1.01 |
|  | M | 53.042 | 51.7 | 5.7 | 89.6 | 0.82 | 0.41 | 1.03 |
| % children with no immunisation | U | 8.8107 | 12.951 | -32.7 |  | -4.12 | 0 | 1.23* |
|  | M | 8.8107 | 7.8233 | 7.8 | 76.1 | 1.28 | 0.201 | 2.60* |

* if variance ratio outside [0.82; 1.21] for M

| Sample | Ps R2 | LR chi2 | p>chi2 | Mean Bias | Med Bias | B | R | %Var |
| --- | --- | --- | --- | --- | --- | --- | --- | --- |
| Unmatched | 0.251 | 228.55 | 0 | 22.2 | 19.9 | 130.7* | 0.69 | 55 |
| Matched | 0.051 | 58.69 | 0 | 8 | 5.3 | 54.7* | 1.09 | 55 |

* if B>25%, R outside [0.5; 2]

Appendix Table A-VII: Mean and standard error (SE) of matching indicator before and after matching

|  |  | Before matching | | After matching | | | |
| --- | --- | --- | --- | --- | --- | --- | --- |
|  |  | Mean | SE | Mean | SE | Mean | SE |
| Region North | control | 0.393 | 0.0300 | 0.556 |  | 0.486 |  |
|  | treat1 | 0.575 | 0.0244 | 0.575 |  |  |  |
|  | treat2 | 0.301 | 0.0286 |  |  | 0.301 |  |
|  | difference treat1-control | 0.182*** | 0.0387 | 0.019 | 0.074 |  |  |
|  | difference treat2-control | -0.092** | 0.0414 |  |  | -0.185** | 0.074 |
| Region West | control | 0.266 | 0.0271 | 0.153 |  | 0.201 |  |
|  | treat1 | 0.172 | 0.0186 | 0.172 |  |  |  |
|  | treat2 | 0.139 | 0.0215 |  |  | 0.139 |  |
|  | difference control-treat1 | -0.094*** | 0.0318 | 0.019 | 0.057 |  |  |
|  | difference control-treat2 | -0.127*** | 0.0347 |  |  | -0.062 | 0.058 |
| Region East | control | 0.037 | 0.0116 | 0.138 |  | 0.143 |  |
|  | treat1 | 0.136 | 0.0198 | 0.136 |  |  |  |
|  | treat2 | 0.517 | 0.0311 |  |  | 0.517 |  |
|  | difference control-treat1 | -0.098*** | 0.0263 | -0.002 | 0.04 |  |  |
|  | difference control-treat2 | 0.480*** | 0.0328 |  |  | 0.375** | 0.05 |
| Region South | control | 0.056 | 0.0141 | 0.017 |  | 0.027 |  |
|  | treat1 | 0.068 | 0.0124 | 0.068 |  |  |  |
|  | treat2 | 0.000 | 0.0000 |  |  | 0.000 |  |
|  | difference control-treat1 | 0.012 | 0.0192 | 0.051 | 0.03 |  |  |
|  | difference control-treat2 | -0.056*** |  |  |  | -0.027* | 0.028 |
| Region Central | control | 0.322 | 0.0287 | 0.444 |  | 0.363 |  |
|  | treat1 | 0.371 | 0.0238 | 0.371 |  |  |  |
|  | treat2 | 0.023 | 0.0094 |  |  | 0.023 |  |
|  | difference control-treat1 | 0.049 | 0.0375 | -0.073 | 0.073 |  |  |
|  | difference control-treat2 | -0.299*** | 0.0305 |  |  | -0.340*** | 0.068 |
| % households with ration card | control | 0.172 | 0.0137 | 0.180 |  | 0.171 |  |
|  | treat1 | 0.169 | 0.0105 | 0.169 |  |  |  |
|  | treat2 | 0.192 | 0.0153 |  |  | 0.192 |  |
|  | difference control-treat1 | -0.003 | 0.0170 | -0.011 | 0.035 |  |  |
|  | difference control-treat2 | 0.020 | 0.0205 |  |  | 0.021 | 0.036 |
| % rural population | control | 0.871 | 0.0121 | 0.891 |  | 0.906 |  |
|  | treat1 | 0.874 | 0.0070 | 0.874 |  |  |  |
|  | treat2 | 0.908 | 0.0091 |  |  | 0.908 |  |
|  | difference control-treat1 | 0.003 | 0.0130 | -0.017 | 0.03 |  |  |
|  | difference control-treat2 | 0.037** | 0.0152 |  |  | 0.002 | 0.023 |
| % SC/ST population | control | 0.482 | 0.0174 | 0.454 |  | 0.442 |  |
|  | treat1 | 0.393 | 0.0104 | 0.393 |  |  |  |
|  | treat2 | 0.507 | 0.0180 |  |  | 0.507 |  |
|  | difference control-treat1 | -0.088*** | 0.0190 | -0.061 | 0.041 |  |  |
|  | difference control-treat2 | 0.025 | 0.0250 |  |  | 0.065 | 0.042 |
| % population illiterate | control | 0.585 | 0.0128 | 0.541 |  | 0.533 |  |
|  | treat1 | 0.529 | 0.0105 | 0.529 |  |  |  |
|  | treat2 | 0.574 | 0.0148 |  |  | 0.574 |  |
|  | difference control-treat1 | -0.056*** | 0.0166 | -0.011 | 0.034 |  |  |
|  | difference control-treat2 | -0.012 | 0.0195 |  |  | 0.040 | 0.033 |
| Primary school per lakh population | control | 96.086 | 0.4696 | 96.255 |  | 96.547 |  |
|  | treat1 | 95.264 | 0.3631 | 95.264 |  |  |  |
|  | treat2 | 93.509 | 0.4919 |  |  | 93.509 |  |
|  | difference control-treat1 | -0.823 | 0.5885 | -0.991 | 1.241 |  |  |
|  | difference control-treat2 | -2.577*** | 0.6797 |  |  | -3.037*** | 0.91 |
| Health Sub centre per lakh population | control | 40.028 | 1.0684 | 39.627 |  | 40.950 |  |
|  | treat1 | 40.479 | 1.0697 | 40.479 |  |  |  |
|  | treat2 | 38.164 | 1.1942 |  |  | 38.487 |  |
|  | difference control-treat1 | 0.451 | 1.5829 | 0.852 | 2.727 |  |  |
|  | difference control-treat2 | -1.863 | 1.6000 |  |  | -2.786** | 2.844 |
| Primary health centre per lakh population | control | 12.965 | 0.8176 | 16.590 |  | 16.174 |  |
|  | treat1 | 15.933 | 1.0104 | 15.933 |  |  |  |
|  | treat2 | 12.958 | 1.1622 |  |  | 12.958 |  |
|  | difference control-treat1 | 2.968** | 1.4174 | -0.658 | 2.265 |  |  |
|  | difference control-treat2 | -0.007 | 1.4140 |  |  | -3.216* | 2.525 |
| Government facility per lakh population | control | 48.047 | 1.2415 | 47.703 |  | 49.706 |  |
|  | treat1 | 47.484 | 1.0915 | 47.484 |  |  |  |
|  | treat2 | 45.642 | 1.3104 |  |  | 45.642 |  |
|  | difference control-treat1 | -0.563 | 1.6845 | -0.219 | 3.138 |  |  |
|  | difference control-treat2 | -2.405 | 1.8041 |  |  | -4.064 | 3.278 |
| Doctor per lakh population | control | 25.663 | 1.4491 | 41.090 |  | 36.646 |  |
|  | treat1 | 41.577 | 1.4757 | 41.577 |  |  |  |
|  | treat2 | 20.529 | 1.2596 |  |  | 20.529 |  |
|  | difference control-treat1 | 15.914*** | 2.1730 | 0.487 | 4.095 |  |  |
|  | difference control-treat2 | -5.134* | 1.9245 |  |  | -16.117*** | 3.733 |
| % children fully immunised | control | 40.122 | 1.4292 | 51.691 |  | 49.666 |  |
|  | treat1 | 53.042 | 1.1583 | 53.042 |  |  |  |
|  | treat2 | 49.690 | 1.3311 |  |  | 49.690 |  |
|  | difference control-treat1 | 12.920*** | 1.8423 | 1.351 | 3.359 |  |  |
|  | difference control-treat2 | 9.568*** | 1.9556 |  |  | 0.024 | 3.347 |
| % children with no immunisation | control | 12.951 | 0.7344 | 7.826 |  | 9.058 |  |
|  | treat1 | 8.811 | 0.6551 | 8.811 |  |  |  |
|  | treat2 | 7.564 | 0.6550 |  |  | 7.564 |  |
|  | difference control-treat1 | -4.140*** | 1.0059 | 0.985 | 1.482 |  |  |
|  | difference control-treat2 | -5.386*** | 0.9859 |  |  | -1.493 | 1.706 |
| # of obs | control | 267 |  | 128 |  | 113 |  |
|  | treat1 | 412 |  | 412 |  |  |  |
|  | treat2 | 259 |  |  |  | 259 |  |

Notes:1. *** significant at 1%, ** significant at 5%, * significant at 10%; 2. significance level of mean difference between treatment and control is assigned using Ho: diff = 0 against the alternative hypothesis Ha: diff != 0

Appendix Table A-VIII: Mean of outcome indicators before and after matching

|  | **% households reporting OOP** | | | **OOP level (INR) per person per month** | | | **% households reporting OOP** | | | **OOP level (INR) per person per month** | | |
| --- | --- | --- | --- | --- | --- | --- | --- | --- | --- | --- | --- | --- |
|  | **IP** | **OP** | **Total** | **IP** | **OP** | **Total** | **IP** | **OP** | **Total** | **IP** | **OP** | **Total** |
| **2000** |  |  |  |  |  |  |  |  |  |  |  |  |
| control | 0.161 | 0.469 | 0.570 | 1.815 | 8.196 | 10.011 | 0.154 | 0.478 | 0.578 | 1.656 | 8.457 | 10.111 |
| treat1 | 0.168 | 0.645 | 0.718 | 2.856 | 14.217 | 17.072 | 0.168 | 0.644 | 0.718 | 2.845 | 14.155 | 16.999 |
| treat2 | 0.183 | 0.532 | 0.637 | 1.5 | 7.878 | 9.378 | 0.183 | 0.532 | 0.637 | 1.50 | 7.879 | 9.38 |
| control-treat1 | -0.007 | -0.176*** | -0.148*** | -1.040*** | -5.771*** | -7.061*** | -0.016** | -0.166*** | -0.140*** | -1.189*** | -5.698*** | -6.887*** |
| control-treat2 | -0.022 | -0.063* | -0.067 | 0.351* | 0.317 | 0.633** | -0.029*** | -0.054*** | -0.059*** | 0.155* | 0.577** | 0.734** |
| **2005** |  |  |  |  |  |  |  |  |  |  |  |  |
| control | 0.066 | 0.510 | 0.544 | 1.059 | 8.397 | 9.456 | 0.082 | 0.521 | 0.560 | 1.129 | 8.296 | 9.425 |
| treat1 | 0.091 | 0.633 | 0.661 | 2.711 | 14.168 | 16.880 | 0.091 | 0.633 | 0.661 | 2.726 | 14.198 | 16.924 |
| treat2 | 0.080 | 0.523 | 0.566 | 1.409 | 8.492 | 9.901 | 0.080 | 0.523 | 0.566 | 1.409 | 8.492 | 9.901 |
| control-treat1 | -0.024*** | -0.122*** | -0.117*** | -1.652*** | -5.771*** | -7.423*** | -0.009* | -0.112*** | -0.101*** | -1.597** | -5.901*** | -7.499*** |
| control-treat2 | -0.014 | -0.013 | -0.022 | -0.35** | 0.095 | -0.445* | -0.001 | -0.002 | -0.006 | -0.280** | -0.196** | -0.476 |
| **2012** |  |  |  |  |  |  |  |  |  |  |  |  |
| control | 0.105 | 0.674 | 0.712 | 2.806 | 13.946 | 16.752 | 0.106 | 0.698 | 0.728 | 2.819 | 14.060 | 16.880 |
| treat1 | 0.137 | 0.804 | 0.830 | 5.254 | 21.231 | 26.485 | 0.138 | 0.804 | 0.831 | 5.277 | 21.225 | 26.502 |
| treat2 | 0.125 | 0.702 | 0.741 | 2.841 | 12.334 | 15.172 | 0.127 | 0.699 | 0.739 | 2.839 | 12.083 | 14.923 |
| control-treat1 | -0.032*** | -0.130*** | -0.119*** | -2.448*** | -7.285*** | -9.732*** | -0.032*** | -0.106*** | -0.103*** | -2.457*** | -7.165*** | -9.622*** |
| control-treat2 | -0.020 | -0.027 | -0.029 | -0.034 | 1.612* ** | 1.578*** | -0.022 | -0.001 | -0.011 | -0.020 | 1.977*** | 1.957*** |
|  | **Before matching** | | | | | | **After matching** | | | | | |
|  | **OOP as share of total household expenditure** | | | **% households reporting OOP share >10% of household expenditure** | | | **OOP as share of total household expenditure** | | | **% households reporting OOP share >10% of household expenditure** | | |
|  | **IP** | **OP** | **Total** | **IP** | **OP** | **Total** | **IP** | **OP** | **Total** | **IP** | **OP** | **Total** |
| **2000** |  |  |  |  |  |  |  |  |  |  |  |  |
| control | 0.005 | 0.021 | 0.026 | 0.008 | 0.046 | 0.060 | 0.004 | 0.021 | 0.026 | 0.008 | 0.046 | 0.059 |
| treat1 | 0.007 | 0.037 | 0.043 | 0.015 | 0.096 | 0.126 | 0.007 | 0.037 | 0.043 | 0.015 | 0.096 | 0.126 |
| treat2 | 0.005 | 0.023 | 0.027 | 0.006 | 0.046 | 0.058 | 0.004 | 0.022 | 0.027 | 0.006 | 0.046 | 0.058 |
| control-treat1 | -0.002 | -0.015*** | -0.018*** | -0.006*** | -0.050*** | -0.066*** | -0.003 | -0.015*** | -0.018*** | -0.007*** | -0.050*** | -0.066*** |
| control-treat2 | 0.000 | 0.001* | 0.001 | 0.002 | 0.000 | 0.002 | 0.000 | -0.001* | -0.001* | -0.002 | 0 | -0.001 |
| **2005** |  |  |  |  |  |  |  |  |  |  |  |  |
| control | 0.002 | 0.021 | 0.024 | 0.007 | 0.046 | 0.056 | 0.003 | 0.021 | 0.024 | 0.007 | 0.046 | 0.056 |
| treat1 | 0.006 | 0.035 | 0.041 | 0.020 | 0.091 | 0.119 | 0.006 | 0.035 | 0.041 | 0.020 | 0.091 | 0.119 |
| treat2 | 0.004 | 0.023 | 0.026 | 0.009 | 0.049 | 0.063 | 0.004 | 0.023 | 0.026 | 0.009 | 0.049 | 0.063 |
| control-treat1 | -0.004** | -0.014*** | -0.017*** | -0.012*** | -0.045** | -0.063*** | -0.003** | -0.014*** | -0.016*** | -0.013*** | -0.045*** | -0.063*** |
| control-treat2 | -0.002** | 0.002* | -0.003** | -0.002* | 0.003 | 0.007* | -0.001* | -0.002** | -0.002*** | -0.002 | -0.003 | -0.007 |
| **2012** |  |  |  |  |  |  |  |  |  |  |  |  |
| control | 0.005 | 0.029 | 0.034 | 0.015 | 0.059 | 0.080 | 0.005 | 0.029 | 0.034 | 0.015 | 0.060 | 0.082 |
| treat1 | 0.010 | 0.041 | 0.051 | 0.03 | 0.101 | 0.150 | 0.010 | 0.041 | 0.051 | 0.030 | 0.101 | 0.150 |
| treat2 | 0.006 | 0.027 | 0.033 | 0.016 | 0.058 | 0.086 | 0.006 | 0.028 | 0.033 | 0.017 | 0.058 | 0.085 |
| control-treat1 | -0.005*** | -0.013* | -0.017*** | -0.015** | -0.042** | -0.069*** | -0.005*** | -0.012*** | -0.016 | -0.015*** | -0.041*** | -0.067*** |
| control-treat2 | -0.001 | 0.002* | 0.001 | -0.001 | 0.001* | 0.005 | -0.001 | -0.001** | -0.001 | -0.002 | 0.002 | -0.003 |

Notes:1. *** significant at 1%, ** significant at 5%, * significant at 10%; 2. significance level of mean difference between treatment and control is assigned using Ho: diff = 0 against the alternative hypothesis Ha: diff != 0
