# Supplementary material for: Extending health insurance to the poor in India: An impact evaluation of Rashtriya Swasthya Bima Yojana on out of pocket spending for healthcare
Source: Soc Sci Med. 2017 May;181:83–92. doi: 10.1016/j.socscimed.2017.03.053 (PMC5408909; doi:10.1016/j.socscimed.2017.03.053)
Supplement: Appendix B [file mmc2.docx]

Appendix Table B-IA: Mean of matching indicators across matched and unmatched sample of treatment and control groups and percentage reduction in bias after matching in the three years – 2011-12, 2004-05 and 1999-2000 (Treatment group ‘treat1’)

|  |  | **2011-12** | | | | | **2004-05** | | | | | **1999-2000** | | | | |
| --- | --- | --- | --- | --- | --- | --- | --- | --- | --- | --- | --- | --- | --- | --- | --- | --- |
| **Treat1** |  | Mean | | % bias | % reduct  bias | p>t | Mean | | % bias | % reduct  bias | p>t | Mean | | % bias | % reduct  bias | p>t |
| Variable |  | Treated | Control |  |  |  | Treated | Control |  |  |  | Treated | Control |  |  |  |
| d_scst | Unmatched | 0.361 | 0.439 | -15.8 |  | 0 | 0.370 | 0.420 | -10.3 |  | 0 | 0.349 | 0.425 | -15.7 |  | 0 |
|  | Matched | 0.371 | 0.362 | 1.8 | 88.4 | 0.158 | 0.373 | 0.372 | 0.2 | 97.7 | 0.822 | 0.357 | 0.347 | 2 | 87.3 | 0.091 |
| d_edu1 | Unmatched | 0.167 | 0.179 | -3.2 |  | 0.012 | 0.236 | 0.274 | -8.6 |  | 0 | 0.345 | 0.405 | -12.4 |  | 0 |
|  | Matched | 0.167 | 0.176 | -2.3 | 27.7 | 0.079 | 0.238 | 0.240 | -0.5 | 94.1 | 0.626 | 0.356 | 0.347 | 1.8 | 85.9 | 0.137 |
| d_edu2 | Unmatched | 0.386 | 0.429 | -8.7 |  | 0 | 0.464 | 0.487 | -4.5 |  | 0 | 0.412 | 0.400 | 2.4 |  | 0.04 |
|  | Matched | 0.394 | 0.388 | 1.2 | 85.7 | 0.348 | 0.468 | 0.472 | -0.9 | 79.5 | 0.384 | 0.413 | 0.412 | 0.1 | 95.8 | 0.933 |
| d_rlgn1 | Unmatched | 0.768 | 0.733 | 8 |  | 0 | 0.772 | 0.715 | 12.9 |  | 0 | 0.764 | 0.761 | 0.8 |  | 0.5 |
|  | Matched | 0.762 | 0.762 | 0.1 | 99.2 | 0.961 | 0.770 | 0.785 | -3.4 | 73.6 | 0.001 | 0.775 | 0.787 | -2.7 | -248.4 | 0.02 |
| d_rlgn2 | Unmatched | 0.144 | 0.128 | 4.6 |  | 0 | 0.133 | 0.169 | -10 |  | 0 | 0.141 | 0.146 | -1.7 |  | 0.152 |
|  | Matched | 0.144 | 0.147 | -0.7 | 83.7 | 0.583 | 0.134 | 0.133 | 0.4 | 95.7 | 0.669 | 0.142 | 0.127 | 4.3 | -159.4 | 0 |
| d_emp1 | Unmatched | 0.279 | 0.304 | -5.5 |  | 0 | 0.267 | 0.260 | 1.4 |  | 0.177 | 0.206 | 0.169 | 9.4 |  | 0 |
|  | Matched | 0.286 | 0.284 | 0.4 | 92.3 | 0.749 | 0.268 | 0.277 | -2.1 | -44.9 | 0.056 | 0.199 | 0.199 | 0 | 99.8 | 0.985 |
| d_emp2 | Unmatched | 0.164 | 0.221 | -14.6 |  | 0 | 0.213 | 0.282 | -16.1 |  | 0 | 0.233 | 0.296 | -14.3 |  | 0 |
|  | Matched | 0.170 | 0.162 | 2.1 | 85.6 | 0.094 | 0.214 | 0.207 | 1.7 | 89.5 | 0.096 | 0.242 | 0.243 | -0.2 | 98.5 | 0.857 |
| d_emp3 | Unmatched | 0.142 | 0.128 | 4.3 |  | 0.001 | 0.059 | 0.048 | 4.8 |  | 0 | 0.048 | 0.048 | 0.1 |  | 0.947 |
|  | Matched | 0.142 | 0.149 | -1.9 | 56.4 | 0.171 | 0.058 | 0.060 | -0.7 | 84.8 | 0.515 | 0.048 | 0.049 | -0.5 | -592.3 | 0.66 |
| d_hhsz | Unmatched | 0.559 | 0.567 | -1.6 |  | 0.204 | 0.510 | 0.506 | 0.9 |  | 0.394 | 0.463 | 0.462 | 0.2 |  | 0.853 |
|  | Matched | 0.556 | 0.550 | 1.2 | 23.7 | 0.351 | 0.511 | 0.504 | 1.3 | -47.5 | 0.214 | 0.461 | 0.471 | -1.9 | -801.2 | 0.108 |
| pr_female | Unmatched | 0.499 | 0.494 | 2.8 |  | 0.027 | 0.500 | 0.495 | 3.2 |  | 0.003 | 0.496 | 0.493 | 1.5 |  | 0.185 |
|  | Matched | 0.497 | 0.497 | -0.2 | 94.7 | 0.909 | 0.500 | 0.501 | -0.4 | 86 | 0.68 | 0.496 | 0.495 | 0.4 | 75.1 | 0.752 |
| pr_married | Unmatched | 0.452 | 0.451 | 0.3 |  | 0.823 | 0.425 | 0.420 | 2.5 |  | 0.019 | 0.418 | 0.419 | -0.4 |  | 0.704 |
|  | Matched | 0.451 | 0.453 | -1 | -244.1 | 0.457 | 0.425 | 0.423 | 0.6 | 77.4 | 0.6 | 0.417 | 0.417 | 0 | 93.9 | 0.982 |
| pr_ag004 | Unmatched | 0.100 | 0.096 | 2.8 |  | 0.028 | 0.118 | 0.121 | -2.4 |  | 0.024 | 0.130 | 0.132 | -1.8 |  | 0.127 |
|  | Matched | 0.100 | 0.101 | -1.3 | 54.6 | 0.34 | 0.118 | 0.119 | -0.7 | 71.4 | 0.522 | 0.132 | 0.128 | 2.1 | -19.5 | 0.079 |
| pr_ag514 | Unmatched | 0.222 | 0.231 | -4.1 |  | 0.001 | 0.254 | 0.258 | -2.2 |  | 0.041 | 0.274 | 0.273 | 0.5 |  | 0.65 |
|  | Matched | 0.226 | 0.223 | 1.2 | 69.8 | 0.347 | 0.255 | 0.256 | -0.6 | 73.9 | 0.599 | 0.275 | 0.276 | -0.2 | 69 | 0.893 |
| pr_ag1529 | Unmatched | 0.259 | 0.263 | -1.6 |  | 0.213 | 0.247 | 0.251 | -1.7 |  | 0.109 | 0.238 | 0.245 | -3.6 |  | 0.002 |
|  | Matched | 0.260 | 0.261 | -0.4 | 74.6 | 0.76 | 0.248 | 0.247 | 0.1 | 94.2 | 0.926 | 0.239 | 0.238 | 0.3 | 90.8 | 0.782 |
| pr_ag60abv | Unmatched | 0.095 | 0.081 | 8 |  | 0 | 0.083 | 0.070 | 7.6 |  | 0 | 0.076 | 0.062 | 9.7 |  | 0 |
|  | Matched | 0.091 | 0.093 | -1 | 87.5 | 0.465 | 0.081 | 0.079 | 1.3 | 83.4 | 0.247 | 0.072 | 0.074 | -1.2 | 87.5 | 0.326 |
| rural1 | Unmatched | 0.715 | 0.746 | -7 |  | 0 | 0.751 | 0.781 | -7.1 |  | 0 | 0.765 | 0.816 | -12.4 |  | 0 |
|  | Matched | 0.717 | 0.708 | 2.1 | 70.7 | 0.128 | 0.753 | 0.746 | 1.7 | 76 | 0.121 | 0.777 | 0.766 | 2.7 | 78.3 | 0.029 |
| safecook | Unmatched | 0.212 | 0.176 | 9.3 |  | 0 | 0.101 | 0.101 | 0.1 |  | 0.925 | 0.102 | 0.106 | -1.3 |  | 0.273 |
|  | Matched | 0.207 | 0.212 | -1.3 | 85.9 | 0.337 | 0.102 | 0.109 | -2.3 | -2182.4 | 0.037 | 0.105 | 0.113 | -2.5 | -94.8 | 0.045 |
| safelight | Unmatched | 0.760 | 0.710 | 11.1 |  | 0 | 0.589 | 0.503 | 17.3 |  | 0 | 0.486 | 0.449 | 7.4 |  | 0 |
|  | Matched | 0.752 | 0.739 | 3 | 73.1 | 0.022 | 0.586 | 0.585 | 0.1 | 99.4 | 0.92 | 0.474 | 0.469 | 1.1 | 85.7 | 0.375 |
| asset_index | Unmatched | 0.274 | 0.259 | 14.9 |  | 0 | 0.099 | 0.093 | 7.7 |  | 0 | 0.043 | 0.045 | -3.7 |  | 0.001 |
|  | Matched | 0.272 | 0.272 | -0.2 | 98.7 | 0.881 | 0.099 | 0.099 | -0.6 | 92.4 | 0.591 | 0.043 | 0.043 | 0.9 | 76.3 | 0.461 |
| bcg | Unmatched | 91.072 | 91.750 | -7.4 |  | 0 | 88.870 | 88.139 | 6.2 |  | 0 | 81.097 | 79.786 | 7.6 |  | 0 |
|  | Matched | 91.503 | 91.647 | -1.6 | 78.8 | 0.212 | 88.803 | 88.560 | 2.1 | 66.8 | 0.054 | 80.531 | 80.650 | -0.7 | 90.9 | 0.576 |
| dpt | Unmatched | 77.583 | 79.245 | -12.9 |  | 0 | 68.340 | 66.439 | 9.5 |  | 0 | 64.950 | 55.818 | 39 |  | 0 |
|  | Matched | 78.096 | 78.045 | 0.4 | 97 | 0.765 | 68.198 | 67.639 | 2.8 | 70.6 | 0.009 | 64.003 | 63.948 | 0.2 | 99.4 | 0.845 |
| polio_3 | Unmatched | 77.436 | 79.295 | -15 |  | 0 | 70.109 | 68.243 | 9.5 |  | 0 | 63.245 | 53.459 | 42.2 |  | 0 |
|  | Matched | 78.127 | 78.190 | -0.5 | 96.6 | 0.702 | 69.982 | 69.456 | 2.7 | 71.8 | 0.012 | 62.258 | 62.126 | 0.6 | 98.6 | 0.638 |
| measles | Unmatched | 78.956 | 81.195 | -17.2 |  | 0 | 72.846 | 70.963 | 10 |  | 0 | 63.706 | 58.118 | 25 |  | 0 |
|  | Matched | 79.911 | 79.826 | 0.6 | 96.2 | 0.616 | 72.732 | 72.323 | 2.2 | 78.3 | 0.041 | 62.817 | 62.874 | -0.3 | 99 | 0.835 |
| vitamin1dose | Unmatched | 65.278 | 61.021 | 24.6 |  | 0 | 60.389 | 59.882 | 2.5 |  | 0.019 | 41.754 | 38.121 | 15.2 |  | 0 |
|  | Matched | 64.850 | 64.337 | 3 | 88 | 0.026 | 60.373 | 60.067 | 1.5 | 39.6 | 0.163 | 41.406 | 40.783 | 2.6 | 82.9 | 0.032 |

Note: * if variance ratio outside [0.95; 1.05] for U and [0.95; 1.05] for M

| Sample | LR chi2 | p>chi2 | MeanBias | MedBias | B | R | %Var |
| --- | --- | --- | --- | --- | --- | --- | --- |
| 2011-12 |  |  |  |  |  |  |  |
| Unmatched | 2548.12 | 0 | 8.6 | 7.7 | 65.6* | 0.97 | 42 |
| Matched | 36.83 | 0.046 | 1.2 | 1.2 | 8 | 0.97 | 58 |
| 2004-05 |  |  |  |  |  |  |  |
| Unmatched | 1114.51 | 0 | 6.6 | 6.7 | 35.6* | 0.86 | 58 |
| Matched | 41.92 | 0.013 | 1.3 | 1.1 | 6.9 | 1.18 | 58 |
| 1999-2000 |  |  |  |  |  |  |  |
| Unmatched | 3858.74 | 0 | 9.5 | 5.6 | 73.6* | 0.69 | 67 |
| Matched | 62.12 | 0 | 1.2 | 0.8 | 9.4 | 0.94 | 67 |

Note: * if B>25%, R outside [0.5; 2]

Appendix Table B-IB: Mean of matching indicators across matched and unmatched sample of treatment and control groups and percentage reduction in bias after matching in the three years – 2011-12, 2004-05 and 1999-2000 (Treatment group ‘treat2’)

|  |  | **2011-12** | | | | | **2004-05** | | | | | **1999-2000** | | | | |
| --- | --- | --- | --- | --- | --- | --- | --- | --- | --- | --- | --- | --- | --- | --- | --- | --- |
| **Treat2** |  | Mean | | %bias | %  reduct  bias | p>t | Mean | | %bias | %  reduct  bias | p>t | Mean | | %bias | %  reduct  bias | p>t |
| Variable |  | Treated | Control |  |  |  | Treated | Control |  |  |  | Treated | Control |  |  |  |
| d_scst | Unmatched | 0.458 | 0.379 | 16 |  | 0 | 0.411 | 0.390 | 4.3 |  | 0 | 0.433 | 0.372 | 12.4 |  | 0 |
|  | Matched | 0.457 | 0.453 | 0.8 | 95.1 | 0.663 | 0.411 | 0.411 | 0 | 99.4 | 0.986 | 0.433 | 0.445 | -2.4 | 80.6 | 0.146 |
| d_edu1 | Unmatched | 0.191 | 0.167 | 6.2 |  | 0 | 0.302 | 0.239 | 14.2 |  | 0 | 0.446 | 0.352 | 19.2 |  | 0 |
|  | Matched | 0.191 | 0.197 | -1.7 | 73.3 | 0.363 | 0.302 | 0.304 | -0.4 | 97 | 0.78 | 0.446 | 0.452 | -1.3 | 93.2 | 0.434 |
| d_edu2 | Unmatched | 0.426 | 0.400 | 5.2 |  | 0 | 0.468 | 0.478 | -2 |  | 0.1 | 0.375 | 0.416 | -8.2 |  | 0 |
|  | Matched | 0.426 | 0.410 | 3.1 | 40.9 | 0.081 | 0.468 | 0.465 | 0.6 | 70.1 | 0.686 | 0.375 | 0.381 | -1.2 | 85.7 | 0.468 |
| d_rlgn1 | Unmatched | 0.697 | 0.770 | -16.5 |  | 0 | 0.728 | 0.748 | -4.6 |  | 0 | 0.776 | 0.758 | 4.2 |  | 0.002 |
|  | Matched | 0.698 | 0.704 | -1.4 | 91.6 | 0.451 | 0.728 | 0.752 | -5.5 | -20 | 0 | 0.776 | 0.765 | 2.5 | 41 | 0.124 |
| d_rlgn2 | Unmatched | 0.141 | 0.134 | 1.8 |  | 0.205 | 0.162 | 0.148 | 3.7 |  | 0.002 | 0.139 | 0.145 | -1.7 |  | 0.214 |
|  | Matched | 0.140 | 0.139 | 0.5 | 73.8 | 0.788 | 0.162 | 0.150 | 3.2 | 14 | 0.029 | 0.139 | 0.132 | 2.1 | -28.1 | 0.183 |
| d_emp1 | Unmatched | 0.320 | 0.281 | 8.6 |  | 0 | 0.287 | 0.255 | 7.2 |  | 0 | 0.181 | 0.189 | -2.1 |  | 0.115 |
|  | Matched | 0.319 | 0.324 | -1.1 | 87 | 0.54 | 0.287 | 0.293 | -1.3 | 82.3 | 0.396 | 0.181 | 0.179 | 0.5 | 77.5 | 0.769 |
| d_emp2 | Unmatched | 0.219 | 0.183 | 9.1 |  | 0 | 0.253 | 0.246 | 1.7 |  | 0.17 | 0.248 | 0.271 | -5.4 |  | 0 |
|  | Matched | 0.218 | 0.224 | -1.3 | 85.5 | 0.473 | 0.253 | 0.249 | 1 | 36.6 | 0.478 | 0.248 | 0.251 | -0.8 | 84.6 | 0.608 |
| d_emp3 | Unmatched | 0.106 | 0.146 | -12 |  | 0 | 0.042 | 0.057 | -7.1 |  | 0 | 0.032 | 0.053 | -10.1 |  | 0 |
|  | Matched | 0.106 | 0.106 | 0.1 | 99.3 | 0.96 | 0.042 | 0.044 | -0.9 | 87.4 | 0.516 | 0.032 | 0.033 | -0.4 | 95.7 | 0.763 |
| d_hhsz | Unmatched | 0.571 | 0.559 | 2.4 |  | 0.097 | 0.524 | 0.503 | 4.2 |  | 0 | 0.496 | 0.452 | 8.9 |  | 0 |
|  | Matched | 0.571 | 0.560 | 2.3 | 6.5 | 0.203 | 0.524 | 0.531 | -1.5 | 63.7 | 0.297 | 0.496 | 0.500 | -0.7 | 91.6 | 0.649 |
| pr_female | Unmatched | 0.496 | 0.497 | -0.1 |  | 0.925 | 0.498 | 0.497 | 0.5 |  | 0.65 | 0.496 | 0.494 | 1.2 |  | 0.348 |
|  | Matched | 0.496 | 0.497 | -0.7 | -383.3 | 0.708 | 0.498 | 0.497 | 0.4 | 20.9 | 0.771 | 0.496 | 0.492 | 2.1 | -68 | 0.202 |
| pr_married | Unmatched | 0.453 | 0.451 | 1.3 |  | 0.379 | 0.419 | 0.424 | -2.5 |  | 0.041 | 0.422 | 0.417 | 2.2 |  | 0.101 |
|  | Matched | 0.453 | 0.453 | 0.3 | 75.9 | 0.864 | 0.419 | 0.421 | -0.9 | 63.1 | 0.535 | 0.422 | 0.422 | -0.2 | 89.8 | 0.892 |
| pr_ag004 | Unmatched | 0.097 | 0.099 | -1.2 |  | 0.394 | 0.123 | 0.118 | 3.4 |  | 0.005 | 0.134 | 0.130 | 2.2 |  | 0.095 |
|  | Matched | 0.097 | 0.097 | -0.4 | 68.2 | 0.823 | 0.123 | 0.124 | -0.4 | 89.4 | 0.81 | 0.134 | 0.133 | 0.5 | 76 | 0.747 |
| pr_ag514 | Unmatched | 0.233 | 0.224 | 4 |  | 0.006 | 0.263 | 0.253 | 4.4 |  | 0 | 0.277 | 0.272 | 2 |  | 0.129 |
|  | Matched | 0.233 | 0.236 | -1.5 | 62.1 | 0.396 | 0.263 | 0.260 | 1.4 | 67.9 | 0.339 | 0.277 | 0.280 | -1.8 | 10.8 | 0.271 |
| pr_ag1529 | Unmatched | 0.258 | 0.262 | -1.8 |  | 0.213 | 0.244 | 0.251 | -2.9 |  | 0.017 | 0.236 | 0.243 | -3.4 |  | 0.01 |
|  | Matched | 0.258 | 0.254 | 1.9 | -3.4 | 0.291 | 0.244 | 0.245 | -0.4 | 87.2 | 0.8 | 0.236 | 0.231 | 2.3 | 34.4 | 0.167 |
| pr_ag60abv | Unmatched | 0.082 | 0.090 | -4.8 |  | 0.001 | 0.068 | 0.080 | -6.6 |  | 0 | 0.063 | 0.071 | -5 |  | 0 |
|  | Matched | 0.082 | 0.084 | -1 | 79.3 | 0.564 | 0.068 | 0.068 | 0.4 | 94.7 | 0.804 | 0.063 | 0.060 | 1.9 | 61.4 | 0.22 |
| rural1 | Unmatched | 0.768 | 0.717 | 11.5 |  | 0 | 0.784 | 0.760 | 5.7 |  | 0 | 0.836 | 0.776 | 15.3 |  | 0 |
|  | Matched | 0.767 | 0.767 | 0.1 | 99 | 0.949 | 0.784 | 0.770 | 3.4 | 40.6 | 0.02 | 0.836 | 0.839 | -0.7 | 95.3 | 0.636 |
| safecook | Unmatched | 0.133 | 0.216 | -21.9 |  | 0 | 0.092 | 0.105 | -4.3 |  | 0 | 0.080 | 0.112 | -10.6 |  | 0 |
|  | Matched | 0.133 | 0.136 | -0.8 | 96.4 | 0.627 | 0.092 | 0.103 | -3.6 | 15.1 | 0.013 | 0.080 | 0.081 | -0.3 | 97.2 | 0.845 |
| safelight | Unmatched | 0.658 | 0.762 | -23.1 |  | 0 | 0.410 | 0.593 | -37.3 |  | 0 | 0.321 | 0.516 | -40.4 |  | 0 |
|  | Matched | 0.660 | 0.642 | 3.8 | 83.5 | 0.043 | 0.410 | 0.415 | -1.1 | 97 | 0.449 | 0.321 | 0.299 | 4.6 | 88.6 | 0.003 |
| asset_index | Unmatched | 0.244 | 0.274 | -29.6 |  | 0 | 0.086 | 0.100 | -18.4 |  | 0 | 0.042 | 0.044 | -4.4 |  | 0.001 |
|  | Matched | 0.245 | 0.241 | 3 | 89.9 | 0.085 | 0.086 | 0.087 | -1.1 | 94 | 0.441 | 0.042 | 0.044 | -2.6 | 41.7 | 0.106 |
| bcg | Unmatched | 90.807 | 91.612 | -7.9 |  | 0 | 88.405 | 88.534 | -1.1 |  | 0.362 | 85.335 | 78.809 | 41.5 |  | 0 |
|  | Matched | 90.819 | 90.526 | 2.9 | 63.5 | 0.117 | 88.405 | 88.227 | 1.5 | -38.2 | 0.325 | 85.335 | 84.178 | 7.4 | 82.3 | 0 |
| dpt | Unmatched | 79.808 | 77.904 | 14.6 |  | 0 | 68.420 | 67.018 | 7.1 |  | 0 | 63.929 | 59.139 | 21.7 |  | 0 |
|  | Matched | 79.785 | 78.552 | 9.4 | 35.2 | 0 | 68.420 | 68.429 | 0 | 99.4 | 0.977 | 63.929 | 62.699 | 5.6 | 74.3 | 0 |
| polio_3 | Unmatched | 79.448 | 77.961 | 11.5 |  | 0 | 68.505 | 69.395 | -4.4 |  | 0 | 62.367 | 56.946 | 25 |  | 0 |
|  | Matched | 79.434 | 78.399 | 8 | 30.4 | 0 | 68.505 | 69.853 | -6.6 | -51.5 | 0 | 62.367 | 61.747 | 2.9 | 88.6 | 0.057 |
| measles | Unmatched | 80.095 | 80.037 | 0.4 |  | 0.761 | 71.513 | 72.027 | -2.7 |  | 0.024 | 63.700 | 59.945 | 18.3 |  | 0 |
|  | Matched | 80.093 | 79.211 | 6.5 | -1425.4 | 0 | 71.513 | 72.976 | -7.6 | -184.4 | 0 | 63.700 | 62.562 | 5.5 | 69.7 | 0 |
| vitAmin1dose | Unmatched | 58.476 | 64.842 | -38.2 |  | 0 | 65.875 | 58.137 | 38.5 |  | 0 | 46.781 | 37.640 | 41.6 |  | 0 |
|  | Matched | 58.546 | 57.602 | 5.7 | 85.2 | 0.002 | 65.875 | 66.577 | -3.5 | 90.9 | 0.016 | 46.781 | 45.374 | 6.4 | 84.6 | 0 |

Note: * if variance ratio outside [0.95; 1.05] for U and [0.95; 1.05] for M

| Sample | LR chi2 | p>chi2 | MeanBias | MedBias | B | R | %Var |
| --- | --- | --- | --- | --- | --- | --- | --- |
| 2011-12 |  |  |  |  |  |  |  |
| Unmatched | 2390.66 | 0 | 10.4 | 8.2 | 71.5* | 1.3 | 50 |
| Matched | 56.52 | 0 | 2.4 | 1.5 | 13.4 | 0.93 | 58 |
| 2004-05 |  |  |  |  |  |  |  |
| Unmatched | 4402.99 | 0 | 7.9 | 4.3 | 83.0* | 0.73 | 58 |
| Matched | 174.42 | 0 | 2 | 1.1 | 19.2 | 2.45* | 50 |
| 1999-2000 |  |  |  |  |  |  |  |
| Unmatched | 3554.45 | 0 | 12.8 | 8.5 | 81.0* | 1.22 | 50 |
| Matched | 89.66 | 0 | 2.4 | 2 | 15.5 | 0.89 | 50 |

Note: * if B>25%, R outside [0.5; 2]

Appendix Table B-II: Mean and standard deviation (error) of outcome indicators across matched treated and matched control sample, average treatment effects and difference-indifference

| Outcomes |  | Treat1 | | | | Treat2 | | | | Diff-in-Diff | | | |
| --- | --- | --- | --- | --- | --- | --- | --- | --- | --- | --- | --- | --- | --- |
|  | Sample | 2011-12 | | 2004-05 | | 2011-12 | | 2004-05 | | Treat1 | | Treat2 | |
|  |  | Mean | SD/SE | Mean | SD/SE | Mean | SD/SE | Mean | SD/SE | Mean | SD/SE | Mean | SD/SE |
| Household expenditure (INR) | Matched treated | 469.441 | 154.116 | 372.498 | 108.7064 | 423.776 | 119.427 | 357.526 | 94.2782 |  |  |  |  |
|  | Matched controls | 437.518 | 143.149 | 372.006 | 105.3750 | 453.190 | 152.794 | 368.184 | 106.7131 |  |  |  |  |
|  | ATT | 31.923 | 1.978 | 0.492 | 1.1840 | -29.415 | 1.930 | -10.658 | 1.2290 | 31.431 | 2.305 | -18.76 | 2.288 |
| % households with any OOP | Matched treated | 0.833 | 0.373 | 0.662 | 0.4731 | 0.737 | 0.440 | 0.566 | 0.4956 |  |  |  |  |
|  | Matched controls | 0.716 | 0.451 | 0.557 | 0.4968 | 0.785 | 0.411 | 0.620 | 0.4855 |  |  |  |  |
|  | ATT | 0.117 | 0.006 | 0.105 | 0.0050 | -0.048 | 0.006 | -0.054 | 0.0060 | 0.012 | 0.008 | 0.006 | 0.008 |
| % households with inpatient OOP | Matched treated | 0.136 | 0.343 | 0.091 | 0.2876 | 0.123 | 0.329 | 0.080 | 0.2719 |  |  |  |  |
|  | Matched controls | 0.108 | 0.311 | 0.071 | 0.2565 | 0.121 | 0.326 | 0.077 | 0.2671 |  |  |  |  |
|  | ATT | 0.028 | 0.004 | 0.020 | 0.0030 | 0.002 | 0.005 | 0.003 | 0.0030 | 0.008 | 0.005 | -0.001 | 0.006 |
| % households with outpatient OOP | Matched treated | 0.808 | 0.394 | 0.633 | 0.4820 | 0.698 | 0.459 | 0.523 | 0.4995 |  |  |  |  |
|  | Matched controls | 0.677 | 0.468 | 0.521 | 0.4996 | 0.755 | 0.430 | 0.591 | 0.4916 |  |  |  |  |
|  | ATT | 0.131 | 0.006 | 0.112 | 0.0050 | -0.057 | 0.007 | -0.068 | 0.0060 | 0.019 | 0.008 | 0.011 | 0.009 |
| Value (INR) of total OOP | Matched treated | 26.520 | 36.917 | 16.924 | 26.5818 | 14.721 | 22.952 | 9.899 | 17.5980 |  |  |  |  |
|  | Matched controls | 15.700 | 24.754 | 9.826 | 17.5534 | 22.859 | 33.988 | 13.776 | 23.1931 |  |  |  |  |
|  | ATT | 10.820 | 0.414 | 7.098 | 0.2450 | -8.138 | 0.394 | -3.877 | 0.2450 | 3.722 | 0.481 | -4.261 | 0.464 |
| Value (INR) of inpatient OOP | Matched treated | 5.178 | 19.692 | 2.726 | 13.1208 | 2.642 | 11.746 | 1.409 | 7.7342 |  |  |  |  |
|  | Matched controls | 2.604 | 12.271 | 1.195 | 6.9969 | 4.093 | 17.133 | 1.967 | 10.7202 |  |  |  |  |
|  | ATT | 2.574 | 0.214 | 1.532 | 0.1140 | -1.451 | 0.202 | -0.557 | 0.1110 | 1.042 | 0.242 | -0.894 | 0.230 |
| Value (INR) of outpatient OOP | Matched treated | 21.343 | 29.896 | 14.198 | 21.9986 | 12.079 | 19.467 | 8.489 | 15.4045 |  |  |  |  |
|  | Matched controls | 13.096 | 20.983 | 8.632 | 15.8341 | 18.767 | 27.979 | 11.809 | 19.7612 |  |  |  |  |
|  | ATT | 8.246 | 0.342 | 5.566 | 0.2090 | -6.688 | 0.329 | -3.320 | 0.2110 | 2.680 | 0.401 | -3.368 | 0.391 |
| Share of total OOP | Matched treated | 0.051 | 0.063 | 0.041 | 0.0590 | 0.033 | 0.049 | 0.026 | 0.0450 |  |  |  |  |
|  | Matched controls | 0.034 | 0.049 | 0.026 | 0.0451 | 0.046 | 0.060 | 0.035 | 0.0546 |  |  |  |  |
|  | ATT | 0.017 | 0.001 | 0.015 | 0.0010 | -0.013 | 0.001 | -0.008 | 0.0010 | 0.002 | 0.001 | -0.005 | 0.001 |
| Share of inpatient OOP | Matched treated | 0.009 | 0.034 | 0.006 | 0.0280 | 0.006 | 0.024 | 0.004 | 0.0190 |  |  |  |  |
|  | Matched controls | 0.005 | 0.024 | 0.003 | 0.0170 | 0.008 | 0.030 | 0.005 | 0.0240 |  |  |  |  |
|  | ATT | 0.004 | 0.000 | 0.003 | 0.0000 | -0.002 | 0.000 | -0.001 | 0.0000 | 0.001 | 0.000 | -0.001 | 0.000 |
| Share of outpatient OOP | Matched treated | 0.042 | 0.053 | 0.035 | 0.0509 | 0.027 | 0.042 | 0.023 | 0.0401 |  |  |  |  |
|  | Matched controls | 0.029 | 0.043 | 0.023 | 0.0413 | 0.038 | 0.051 | 0.030 | 0.0482 |  |  |  |  |
|  | ATT | 0.013 | 0.001 | 0.012 | 0.0010 | -0.011 | 0.001 | -0.007 | 0.0010 | 0.001 | 0.001 | -0.004 | 0.001 |
| % households with catastrophic total OOP | Matched treated | 0.151 | 0.358 | 0.119 | 0.3243 | 0.085 | 0.279 | 0.063 | 0.2423 |  |  |  |  |
|  | Matched controls | 0.087 | 0.282 | 0.062 | 0.2411 | 0.130 | 0.336 | 0.096 | 0.2945 |  |  |  |  |
|  | ATT | 0.064 | 0.004 | 0.057 | 0.0030 | -0.044 | 0.004 | -0.033 | 0.0030 | 0.007 | 0.005 | -0.011 | 0.005 |
| % households with catastrophic inpatient OOP | Matched treated | 0.030 | 0.170 | 0.020 | 0.1388 | 0.016 | 0.125 | 0.009 | 0.0966 |  |  |  |  |
|  | Matched controls | 0.015 | 0.121 | 0.008 | 0.0894 | 0.024 | 0.153 | 0.015 | 0.1208 |  |  |  |  |
|  | ATT | 0.015 | 0.002 | 0.012 | 0.0010 | -0.008 | 0.002 | -0.005 | 0.0010 | 0.003 | 0.002 | -0.003 | 0.002 |
| % households with catastrophic outpatient OOP | Matched treated | 0.102 | 0.303 | 0.091 | 0.2876 | 0.060 | 0.238 | 0.049 | 0.2154 |  |  |  |  |
|  | Matched controls | 0.064 | 0.245 | 0.050 | 0.2181 | 0.092 | 0.289 | 0.075 | 0.2631 |  |  |  |  |
|  | ATT | 0.038 | 0.004 | 0.041 | 0.0030 | -0.032 | 0.004 | -0.026 | 0.0030 | -0.003 | 0.005 | -0.006 | 0.005 |
| Number of observations | Matched treated | 12523 |  | 17713 |  | 6321 |  | 9,235 |  |  |  |  |  |
|  | Matched controls | 12005 |  | 18,088 |  | 18089 |  | 26,106 |  |  |  |  |  |

Appendix Table B-III: Effects of RSBY on inpatient, outpatient and total OOP with matched household sample

|  | Inpatient | | | | Outpatient | | | | Total OOP | | | |
| --- | --- | --- | --- | --- | --- | --- | --- | --- | --- | --- | --- | --- |
|  | Probability of any OOP | OOP Level (INR) | OOP Share | Probability of Catastrophic | Probability of any OOP | OOP Level (INR) | OOP Share | Probability of Catastrophic | Probability of any OOP | OOP Level (INR) | OOP Share | Probability of Catastrophic |
| **‘treat1’ Districts** | | | | | | | | | | | | |
| t2_treat1 | 1.344 | 0.048 | 0.007 | 1.598* | 0.814* | 0.048 | 0.003 | 1.019 | 0.847 | 0.014 | 0.004 | 1.084 |
| SE | 0.2647 | 0.1433 | 0.0048 | 0.3895 | 0.0848 | 0.0508 | 0.0022 | 0.1227 | 0.0818 | 0.0581 | 0.0025 | 0.1250 |
| t3_treat1 | 1.475** | 0.033 | 0.004 | 1.239 | 1.039 | -0.014 | -0.001 | 0.832 | 1.096 | -0.029 | 0.000 | 0.945 |
| SE | 0.2783 | 0.1131 | 0.0042 | 0.2599 | 0.1315 | 0.0565 | 0.0026 | 0.1053 | 0.1426 | 0.0593 | 0.0026 | 0.1030 |
| *Ratio of odds ratios or differences in pre- and post-intervention DID coefficients* | | | | | | | | | | | | |
| t3_treat1-  t2_treat1 | 1.097 | 0.016 | -0.003 | 0.776 | 1.277** | -0.062 | -0.004 | 0.817 | 1.294* | -0.042 | -0.003 | 0.871 |
| SE | 0.2043 | 0.1202 | 0.0049 | 0.1853 | 0.1565 | 0.0588 | 0.0025 | 0.1116 | 0. 1622 | 0.0567 | 0.0026 | 0.1093 |
| **‘treat2’ Districts** | | | | | | | | | | | | |
| t2_treat2 | 1.036 | 0.390* | 0.010** | 1.882** | 0.797* | 0.120* | 0.005 | 1.114 | 0.786 | 0.128* | 0.007** | 1.243 |
| SE | 0.2421 | 0.1498 | 0.0048 | 0.5510 | 0.0898 | 0.0648 | 0.0029 | 0.2039 | 0.0890 | 0.0708 | 0.0030 | 0.2149 |
| t3_treat2 | 1.187 | 0.240 | 0.007 | 2.066*** | 0.854 | -0.036 | 0.002 | 1.051 | 0.812 | 0.017 | 0.005* | 1.264 |
| SE | 0.2698 | 0.1231 | 0.0046 | 0.5721 | 0.1182 | 0.0676 | 0.0029 | 0.1895 | 0.1168 | 0.0687 | 0.0029 | 0.2037 |
| *Ratio of odds ratios or differences in pre- and post-intervention DID coefficients* | | | | | | | | | | | | |
| t3_treat1-  t2_treat1 | 1.145 | -0.150 | -0.003 | 1.097 | 1.071 | -0.156** | -0.028 | 0.943 | 1.033 | -0.110* | -0.002 | 1.017 |
| SE | 0.2484 | 0.1333 | 0.0053 | 0.3285 | 0.1489 | 0.0740 | 0.0029 | 0.1675 | 0.1404 | 0.0735 | 0.0032 | 0.1738 |
|  |  |  |  |  |  |  |  |  |  |  |  |  |
| R-2/pseudo-R2 | 0.079 | 0.31 | 0.15 | 0.071 | 0.093 | 0.140 | 0.07 | 0.058 | 0.085 | 0.157 | 0.084 | 0.071 |
| Observations | 90501 | 10984 | 10984 | 90501 | 90501 | 56212 | 56212 | 90501 | 90501 | 60770 | 60770 | 90501 |

Notes: 1. * significant at 10% level; ** significant at 5% level; *** significant at 1% level; 2. standard errors in are mentioned in the second row against each co-efficient/odds ratio; 3. Standard errors clustered at village level; 4. Values in the probability columns are odds ratios of the probabilities of incurring any OOP and catastrophic payments and under OOP level and OOP share are coefficients of per person monthly OOP and OOP expenditure as a share of households’ total consumption expenditure respectively. 5. Values under OOP share should be multiplied with 100 to read in percentage terms.
